# Multi-objective optimization reveals time- and dose-dependent inflammatory cytokine-mediated regulation of human stem cell derived T-cell development

**DOI:** 10.1038/s41536-022-00210-1

**Published:** 2022-01-27

**Authors:** John M. Edgar, Yale S. Michaels, Peter W. Zandstra

**Affiliations:** 1 School of Biomedical Engineering, University of British Columbia, Vancouver, BC V6T 1Z3 Canada; 2 Michael Smith Laboratories, University of British Columbia, Vancouver, BC V6T 1Z4 Canada

**Keywords:** Stem-cell biotechnology, Translational immunology, Haematopoietic stem cells, Extracellular signalling molecules, Stem-cell differentiation

## Abstract

The generation of T-cells from stem cells in vitro could provide an alternative source of cells for immunotherapies. T-cell development from hematopoietic stem and progenitor cells (HSPCs) is tightly regulated through Notch pathway activation by Delta-like (DL) ligands 1 and 4. Other molecules, such as stem cell factor (SCF) and interleukin (IL)-7, play a supportive role in regulating the survival, differentiation, and proliferation of developing T-cells. Numerous other signaling molecules influence T-lineage development in vivo, but little work has been done to understand and optimize their use for T-cell production. Using a defined engineered thymic niche system, we undertook a multi-stage statistical learning-based optimization campaign and identified IL-3 and tumor necrosis factor α (TNFα) as a stage- and dose-specific enhancers of cell proliferation and T-lineage differentiation. We used this information to construct an efficient three-stage process for generating conventional TCRαβ^+^CD8^+^ T-cells expressing a diverse TCR repertoire from blood stem cells. Our work provides new insight into T-cell development and a robust system for generating T-cells to enable clinical therapies for treating cancer and immune disorders.

## Introduction

T-cell immunotherapies using cancer antigen-specific T-cell receptors (TCRs) or chimeric antigen receptors (CARs) have emerged as a potent treatment option for diseases such as B-cell acute lymphoblastic leukemia (B-ALL) and diffuse large B-cell lymphoma^1^. These therapies collect peripheral T-cells from patients and expand and transduce them with a CAR before returning the CAR T-cells back to the patient. While these therapies are effective, the reliance on patient-derived cells is a limitation, as patients undergoing chemotherapy may not have an adequate number of cells for CAR T-cell therapy. Additionally, in vitro expansion can lead to T-cell exhaustion, limiting long-term efficacy^2^. The generation of T-cells from stem cells in vitro could provide an alternative source of cells for immunotherapies. Stem cells could be genetically engineered using CRISPR/Cas9 to target a CAR to the TCR α constant (TRAC) locus, as was done previously with peripheral T-cells, to provide CAR expression dynamics that mimic physiological TCR expression and enable better therapeutic cell products^3^. However, current methods for generating T-cells in vitro are impeded by low efficiency and often produce mixed populations of T, natural killer (NK), and myeloid lineage cells. To generate T-cells in sufficient quantities for therapies, new strategies are needed to enhance overall cell yield while maintaining a relatively pure population of T-cells.

T-cell development in vivo occurs in the thymus. Hematopoietic progenitor cells migrate from bone marrow to the thymus where they initiate T-lineage development^4,5^. Upon entry into the thymus, progenitors quickly upregulate CD7 and are defined as CD4/8 double negative (DN) CD34^+^CD7^+^(CD5^−^) proT1^6^. Throughout T-lineage specification, cells develop to become CD34^+^CD7^+^CD5^+^ proT2. Here, recombination of the TCRβ locus is initiated and is associated with T-lineage commitment. Cells begin to express CD4, passing through a CD4^+^ immature single-positive (CD4ISP) stage before becoming CD4/8 double-positive (DP)^7^. The TCRα locus is recombined, and cells undergo positive selection to ensure productive rearrangements. Cells lose expression of CD4 or CD8, depending on the specificity of the TCRαβ for a particular human leukocyte antigen (HLA) allele, and begin negative selection to eliminate potentially autoreactive T-cells. Those that pass all selection checkpoints enter the periphery as CD4^+^CD8^−^ or CD4^−^CD8^+^ single-positive (SP) T-cells^8^.

The initiation of T-lineage development is driven by Notch1 pathway activation through exposure to ligands Delta-like (DL) 1 and 4 and Jagged-2 expressed on the surface of thymic epithelial cells^9,10^. The protein vascular cell adhesion molecule 1 (VCAM-1) is co-expressed with Notch ligands on TECs surfaces, where it facilitates migration through the thymus and continuous exposure to Notch ligands^11^. Other molecules present in the thymus, including stem cell factor (SCF) and interleukin (IL)-7, supplement Notch1 signaling to support T-cell survival, differentiation, and proliferation. Many other signaling molecules are present in the thymus, including IL-1α/β, IL-3, IL-6, IL-12, and tumor necrosis factor α (TNFα)^12–14^, although their functions in human T-cell development are not as well understood.

A number of methods exist for generating T-cells in vitro^15–17^. One of the most successful methods involves co-culturing hematopoietic stem and progenitor cells (HSPCs) with mouse OP9 bone marrow stromal cells that ectopically express the Notch ligand DL1 or DL4 (OP9-DL)^16^. This system is robust and able to support development throughout proT and DP stages to generate CD8SP T-cells^18,19^. Other approaches have replaced OP9-DL with recombinant DL4-Fc fusion proteins immobilized to surfaces or microbeads^20,21^. Both serum and serum-free versions have been described with mixed success, and these are often limited to the early stages of T-lineage specification.

We have previously described a defined engineered thymic niche (ETN) system for generating proT-cells from human umbilical cord blood (UCB)-derived CD34^+^ HSPCs^22^. This system uses plate-bound DL4-Fc and VCAM-1-Fc along with the recombinant cytokines SCF, FMS-like tyrosine kinase 3 ligand (Flt3L), IL-7, and thrombopoietin (TPO) in a serum-free medium to facilitate proT-cell development. While it generates proT-cells with similar efficiency to OP9-DL cocultures, this system provides only the minimum essential components necessary to do so. However, it does not support T-cell maturation which is a key step for clinical implementation. We hypothesized that incorporating additional thymus-associated cytokines into ETN would enhance T-lineage differentiation and proliferation. Through a targeted cytokine screen, we identified IL-3 and TNFα as enhancers of T-lineage development. Mechanistically, we found that TNFα accelerates T-lineage specification through interactions with the Notch1 signaling pathway. Combining TNFα with IL-3 resulted in a significant expansion of proT-cells with minimal myeloid and NK cell contamination. We then used sequential multifactor models to optimize the stage-specific cytokine requirements necessary for T-cell maturation and show that TNFα switches from a differentiation enhancer to inhibitor during T-cell development. We provide a platform strategy for quantitively predicting dynamic cytokine signaling requirements in developmental processes and a three-stage ETN system for generating DP and CD8^+^ T-cells in a defined, scalable, and clinically-relevant manner.

## Results

### A two-phase screening strategy for enhancers of ProT-cell differentiation

**Fig. 1:**
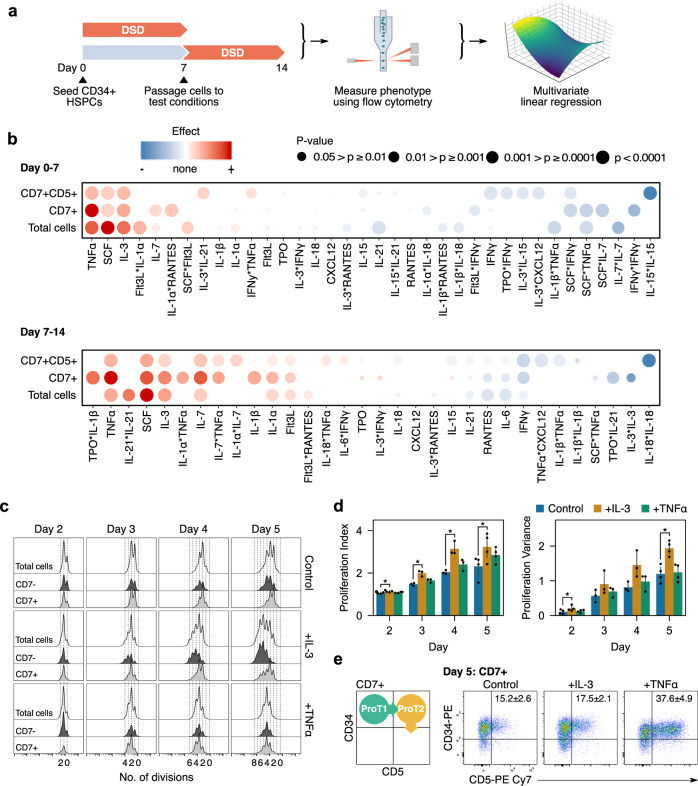
Screening for cytokines that enhance proT-cell differentiation and expansion. **a** Summary of two-part screening experiment workflow. Cells were cultured in screening conditions from day 0–7 or cultured until day 7 and passaged at equal densities into test conditions and cultured until day 14. Cells from day 7 and 14 were harvested and analyzed using flow cytometry. The absolute count of each population of interest was measured and used to calculate a *z*-score relative to the 4F control. The *z*-scores were then used to fit multivariate linear regression models. **b** Effect of cytokines on total cells, CD7^+^ lymphocytes, and CD7^+^CD5^+^ cells. Red indicates an effect greater than the control condition while blue indicates an effect lesser than the control. The size of the circle indicates the significance of the effect in the regression model. From *n* = 2 independent UCB donors. **c** Histograms of CFSE stained cells showing the number of divisions of each cell on days 2–5. Separation of CD7^−^ and CD7^+^ histograms show differential responses to each cytokine. **d** Proliferation statistics from CFSE data. IL-3 treated cells had a larger proliferative index indicating that they underwent, on average, more divisions than the control group. They also had a significantly larger proliferative indicating that some cells responded much stronger to IL-3 than others. *p < 0.05 relative to the control on each day. **e** All groups transitioned through a proT1 to proT2 phenotype as expected during T-cell development. Results are mean ± standard error from *n* = 4 independent UCB donors.

Using the DL4 + VCAM-1 ETN platform, we sought to identify soluble cytokines that positively regulate T-lineage differentiation and expansion from UCB-derived CD34^+^ HSPCs (Supplementary Fig. 1). A list of 15 candidate molecules was assembled from a survey of T-cell development literature in both mouse and human. These molecules were tested in combination at three concentrations each for total, CD7^+^ lymphocyte, and CD7^+^CD5^+^ proT-cell expansion. To separate the effects of cytokines on early HSPCs and emerging proT-cells, the experiment was performed in two separate stages (Fig. 1a). Test conditions were compared to a control condition that contained SCF, Flt3L, TPO, and IL-7 (4F) at 100 ng/ml each, as was used in our previous work^22^.

Of the 15 cytokines tested, SCF, IL-3, and TNFα elicited strong proliferative effects from days 0–7 on all three populations, while IL-7 had only a small effect on total and CD7^+^ cell numbers (Fig. 1b). From day 7–14, the effect of IL-7 was much greater, although cells still responded most strongly to SCF, IL-3, and TNFα. Other cytokines, such as IFNγ and IL-6, had a negative effect on the expansion of one or more of the cell populations and were excluded from future experiments. A similar experiment was performed including IL-3 and TNFα at a higher range of concentrations to confirm our observations (Supplementary Fig. 2). Based on this experiment, working concentrations for IL-3 and TNFα were chosen as 10 and 5 ng/ml, respectively.

We measured cell proliferation using carboxyfluorescein succinimidyl ester (CFSE) dye for 4F cytokines (control) or 4F plus one of IL-3 and TNFα. Cells treated with IL-3 proliferated more than the control, but this was primarily in the non-lymphoid (CD7^-^) fraction (Fig. 1c, d). Cells treated with TNFα proliferated similarly to the control. All cells transitioned through proT1 and proT2 stages after 5 days, consistent with development on OP9-DL4^6^. However, a significantly (*p* < 0.05) higher proportion of CD7^+^ cells treated with TNFα had a proT2 phenotype (37.6 ± 4.9%) compared to the IL-3 (17.5 ± 2.1%) and control (15.2 ± 2.6%) (Fig. 1e).

### Interactions between TNFα and the Notch pathway enhances T-lineage differentiation

**Fig. 2:**
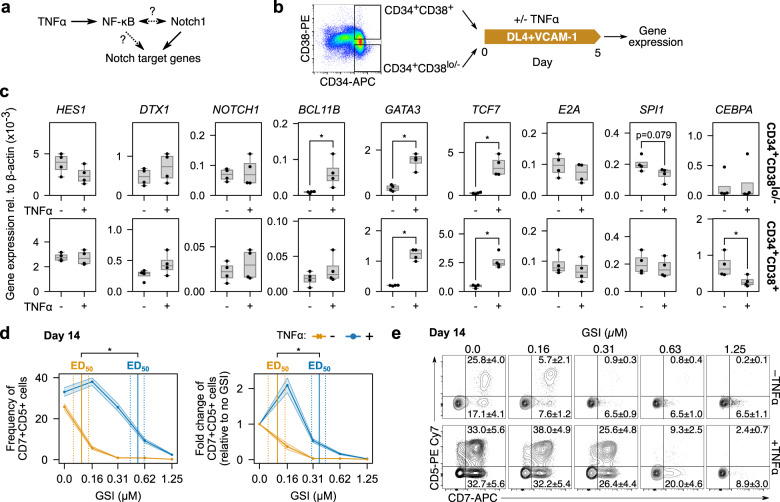
Synergy between TNFα and the Notch pathway compensates for low Notch activation. **a** TNFα activates the NF-κB pathway, which may regulate Notch target genes or regulate Notch itself. **b** To investigate the ways that TNFα may be interacting with Notch, CD34^+^ HSPCs were sorted into CD38^lo/−^ and CD38^+^ fractions and seeded separately on DL4 + VCAM-1 with and without TNFα. Gene expression was measured using qPCR after 5 days of culture. **c** Both the CD38^lo/−^ and CD38^+^ fractions upregulated *GATA3* and *TCF7* in response to TNFα while only CD38^lo/−^ HSPCs upregulated *BCL11B*. No differences were observed in any other Notch target genes, implying that TNFα is not regulating Notch itself. CD38^lo/−^ but not CD38^+^ HSPCs downregulated *SPI1* slightly in response to TNFα. In contrast, only the CD38^+^ fraction significantly downregulated *CEBPA* when cultured with TNFα. Bar plots show a median and interquartile range of *n* = 4 independent UCB donors. **d** CD34^+^ HSPCs were seeded on DL4 + VCAM-1 for 14 days with increasing concentrations of γ-secretase inhibitor (GSI) to inhibit Notch activation. TNFα was able to maintain CD7^+^CD5^+^ cell generation with a significantly higher concentration of GSI than without. **e** Representative flow cytometry plots show the differential effects of Notch inhibition with and without TNFα. **d**, **e** are mean ± standard error from *n* = 7 independent UCB donors and *p < 0.05.

The early increased proportion of a proT2 phenotype in cells treated with TNFα made us ask whether it was interacting with the Notch1 pathway to enhance T-lineage specification. TNFα signals through the NF-κB pathway, which, in other cell types, has been shown to interact extensively with Notch in a context-dependent manner (Fig. 2a)^23^. We were interested to see if the effects of TNFα were specific to HSCs and multipotent progenitors (MPPs) or their downstream progeny. We therefore sorted CD34^+^ HSPCs into CD38^lo/-^ and CD38^+^ fractions to separate HSCs/MPPs from more differentiated progenitors. We seeded each fraction on DL4 + VCAM-1 with and without TNFα and measured the expression of Notch target genes after 5 days (Fig. 2b). The addition of TNFα increased the expression of *GATA3* and *TCF7* (encoding TCF-1)—genes that are important for T-lineage specification^24^—in both the CD38^lo/−^ and CD38^+^ fractions (Fig. 2c). *BCL11B*, a gene associated with T-lineage specification and commitment^25^, was also upregulated in CD38^lo/−^ cells treated with TNFα. No significant differences were observed in *HES1*, *DTX1*, *E2A*, or *NOTCH1* mRNA levels, suggesting that this effect was not due to an increase in Notch1 receptor expression and overall Notch pathway activation. TNFα also induced a modest decrease in the myeloid gene *SPI1* (encoding PU.1) in CD38^lo/−^ HSPCs, and a significant decrease in *CEBPA* in CD38^+^ HSPCs. The decrease in *CEBPA* mRNA levels was not due to an increase in *HES1* expression, which antagonizes *CEBPA*^26^. The upregulation of T-lineage genes and decrease in pro-myeloid-lineage genes by TNFα provides a mechanism by which it inhibits myeloid differentiation. The increased expression of *BCL11B* in only the CD38^lo/-^ fraction implies that they have a higher propensity for T-lineage differentiation, consistent with the previous reports^6^.

Given that TNFα enhances the expression of T-lineage specification genes, we next tested whether it could decrease dependence on Notch signaling during T-cell development. We placed CD34^+^ HSPCs on DL4 + VCAM-1 for 14 days and used the γ-secretase inhibitor (GSI) DAPT to inhibit Notch activation. We calculated the 50% effective dose (ED_50_) of GSI for CD7^+^CD5^+^ cells with and without TNFα using linear interpolation. Without TNFα, the ED_50_ for the frequency of CD7^+^CD5^+^ cells was 0.12 ± 0.02 µM (mean ± standard error); with TNFα, the ED_50_ was 0.52 ± 0.10 µM (Fig. 2d, e). Similarly, the ED_50_ for the fold-change in CD7^+^CD5^+^ cell numbers without TNFα was 0.12 ± 0.03 µM and with TNFα was 0.36 ± 0.06 µM. TNFα was able to maintain CD7^+^CD5^+^ cell generation with a significantly higher concentration of GSI than without. Thus, although it cannot replace Notch activity completely, TNFα can partially compensate for Notch pathway inhibition through the regulation of Notch target genes.

### TNFα synergizes with IL-3 to enhance ProT-cell expansion

Cells treated with only IL-3 divided more than those not treated with IL-3. However, increased proliferation was observed predominantly in the CD7^-^ non-lymphoid population. This finding contrasted with our screening experiment that found that IL-3 stimulated the proliferation of CD7^+^ lymphoid cells. Because our screen tested all factors in combination, we hypothesize that IL-3 may be interacting synergistically with TNFα. To test this, we seeded CD34^+^ HSPCs on DL4 + VCAM-1 with IL-3+TNFα in combination. The use of both cytokines led to significantly higher expansion than with any single cytokine alone, and cells were confluent by day 7 and required passaging.

**Fig. 3:**
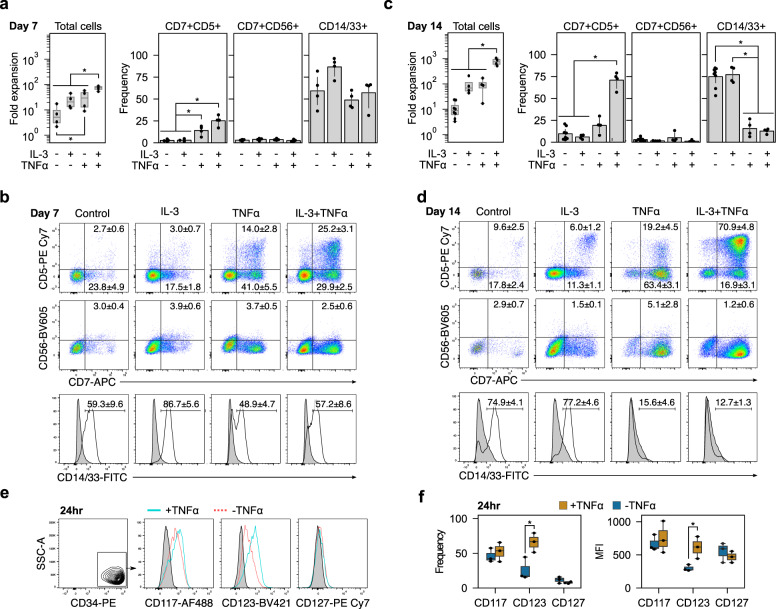
IL-3 and TNFα enhance proT-cell expansion and purity. CD34^+^ HSPCs were placed on DL4 + VCAM-1 and fold expansion and phenotype were measured on days 7 and 14. **a** Fold expansion and frequency of CD7^+^CD5^+^ proT, CD7^+^CD56^+^ NK, and CD14/33^+^ myeloid cells on day 7. Combining TNFα with IL-3 significantly increased total cell expansion over all other conditions. It also increased the frequency of CD7^+^CD5^+^ cells without increasing CD7^+^CD56^+^ frequencies. Box plots show median and interquartile range from *n* = 4 independent UCB donors and bar plots are mean ± standard error (*p < 0.05). **b** Representative flow cytometry plots on day 7. Frequencies are mean ± standard error. **c** By day 14, the frequency of CD14/33^+^ cells was significantly lower in groups containing TNFα than those without. Box plots show median and interquartile range from *n* = 4 independent UCB donors and bar plots are mean ± standard error (*p < 0.05). **d** Representative flow cytometry plots from day 14 show a relatively pure population of CD7^+^CD5^+^ cells when TNFα is combined with IL-3. Frequencies are mean ± standard error. **e** Representative flow cytometry plots of CD117, CD123, and CD127 expression on CD34^+^ HSPCs with or without TNFα stimulation for 24 h. **f** TNFα induced a significant increase in the frequency of CD123^+^ cells. The increased frequency of CD123^+^ cells was accompanied by an increase in the median fluorescent intensity (MFI) of CD123, indicating a higher receptor density on the cell’s surface after TNFα stimulation. *p < 0.05 for *n* = 3 independent UCB donors.

By day 7, the IL-3+TNFα group had expanded 75.0 (55.6–86.8)-fold (median, 5–95 percentile) compared to 20.7 (12.2–42.7)-fold in IL-3, 29.5 (9.74–55.9)-fold in TNFα, and 5.1 (2.4–15.9)-fold in the control groups (Fig. 3a, b). They also had a higher frequency of CD7^+^CD5^+^ cells than the IL-3 and control groups. CD7^+^CD56^+^ NK frequencies were less than 4% in all groups, and the frequencies of CD14/33^+^ myeloid cells were variable and not significantly different. By day 14, fold expansion was an order of magnitude greater in the IL-3+TNFα group at 753.2 (532.4–1026.9)-fold, compared to 69.0 (45.8–190.8)-fold in the IL-3, 90.7 (27.5–159.1)-fold in the TNFα, and 8.9 (4.3–21.5)-fold in the control groups (Fig. 3c, d). The frequency of CD7^+^CD5^+^ cells was also significantly greater with IL-3+TNFα than all other conditions. As on day 7, CD7^+^CD56^+^ NK frequencies were low, and both TNFα and IL-3+TNFα groups had a significantly lower frequency of CD14/33^+^ myeloid than IL-3 or control groups. Thus, IL-3+TNFα synergize to elicit significant proliferation and preferentially enrich cultures for CD7^+^CD5^+^ cells.

### TNFα regulates expression of the IL-3 receptor

To elucidate a mechanism for the synergistic effect of IL-3 and TNFα, we examined the expression of the IL-3 receptor (CD123) after stimulation of CD34^+^ HSPCs with TNFα (Fig. 3e). TNFα has been reported to upregulate CD123 in human bone marrow (BM)-derived CD34^+^ HSPCs^27^. Consistent with this, we found that TNFα increased the frequency of CD123^+^ cells after 24 h (Fig. 3f). TNFα stimulation also increased the median fluorescent intensity (MFI) of CD123 expression, indicating an increase in the number of CD123 molecules on the surface of cells. We observed no change in the frequency and MFI of the SCF (CD117) and IL-7 (CD127) receptors in our UCB-derived HSPCs. The synergy between IL-3 and TNFα is therefore due, at least in part, to increased responsiveness of cells to IL-3 through an increase in the number of cells expressing the receptor as well as an increase in the level of expression.

### TNFα switches from an enhancer to an inhibitor during T-cell development

**Fig. 4:**
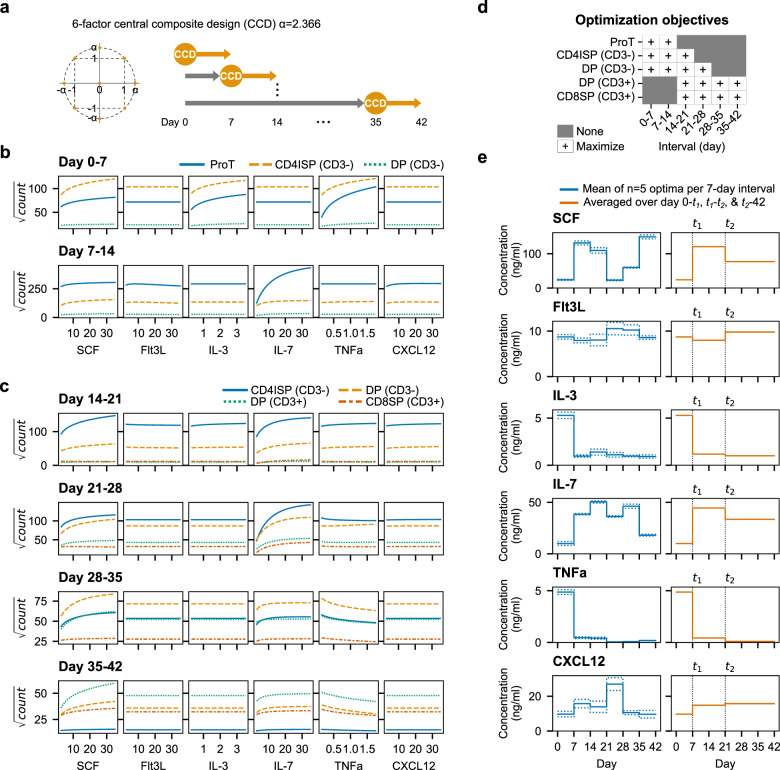
SCF and IL-7 promote T-cell proliferation while TNFα inhibits maturation. **a** The RSM was constructed as a six-factor central composite design. Experiments were performed in 7-day intervals until day 42 to measure cytokine responses during all stages of T-cell development. **b** Cytokine dose responses for proT, CD4ISP, and early DP (CD3^-^) between days 0–7 and 7–14. Cells were unresponsive to IL-7 until day 7–14 but respond strongly to SCF, IL-3, and TNFα from day 0–7. **c** Cytokine dose responses for CD4ISP, early DP, and late DP (CD3^+^), and CD8SP for each 7-day interval between days 14–42. The positive dose-dependent effect of IL-3 and TNFα early in cultures flattens and TNFα begins to inhibit the generation of DP and CD8SP cells. In (**b**, **c**), cytokine concentrations were swept from low to high while holding all other cytokines at their scaled center value (0). Shown are square-root transformed cell counts for each population. Experiments used *n* = 3 pooled UCB donors. **d** Objectives for optimization of RSM. Populations were either maximized or not included/present in certain 7-day intervals. **e** Optimized cytokines per 7-day interval (left) or as a three-stage assay (right). Solid lines are the mean of the top five optimizations while dotted lines represent the standard deviation.

Next, we sought to identify cytokine signaling requirements for T-lineage maturation on DL4 + VCAM-1. We used response surface methodology (RSM) to model the dose-response of cells to SCF, Flt3L, IL-3, IL-7, TNFα, and CXCL12 (Fig. 4a). Multivariate regression was used to fit polynomial models to experimental data (Supplementary Fig. 3). We excluded TPO after we found that removing it reduced CD14/33^+^ myeloid generation without detrimentally affecting CD7^+^ lymphoid expansion (Supplementary Fig. 4). CXCL12 was included because we observed a modest but positive effect in screening experiments and because of its reported positive effect on cell survival during β-selection^28,29^. Following our Notch inhibition experiment and reports that αβT-cell development requires reduced Notch pathway activation around the β-selection checkpoint^30^, we titrated DL4 and reduced the concentration 7.5-fold while maintaining similar proportions of proT-cells (Supplementary Fig. 5). Experiments were conducted over 7-day intervals, and the number of cells in each population was measured using flow cytometry. Because the RSM has higher predictive power than the definitive screening design, we included the first 2 weeks of differentiation to estimate cytokine dose responses more accurately during T-cell specification. We measured proT-cells, CD4ISPs, and early DPs (CD3^−^) during the first 14 days. From day 14 onwards, we included CD4ISPs, early DPs, late DPs (CD3^+^), and CD8SPs (CD4^−^CD8α^+^CD3^+^).

On day 7, large populations of proT and CD4ISP cells were present in cultures. These cells responded strongly to increasing concentrations of SCF, IL-3, and TNFα (Fig. 4b). Positive two-factor interactive effects were observed between SCF and TNFα and IL-3 and TNFα (Supplementary Fig. 6). A small number of early DP cells was also present by day 7. Between days 7–14, all populations were responsive to increasing concentrations of SCF and IL-7 and unresponsive to IL-3. Cells responded positively to TNFα and CXCL12 with a negative interaction, but the magnitude of these effects was small compared to SCF and IL-7 (Supplementary Fig. 6).

Cultures were primarily CD3^−^ on day 21, but the proportions of CD3^+^ cells steadily increased from day 28 onwards (Fig. 4c). Again, TNFα had a small positive effect on cell expansion at higher concentrations between day 14–21, but this became negative from day 21 onwards. Differentiation was dominated by SCF and IL-7, and the cytokines had some interactive effects from day 21 through day 35 (Supplementary Fig. 6). The response to IL-3 was small between day 21–28; cells were completely unresponsive to IL-3 from day 28 onwards. Flt3L had little or no measurable effect on cell expansion throughout the entire assay.

### An optimized three-stage process for T-cell generation from blood stem cells

In order to define a set of preferred conditions for each step in the differentiation, we optimized the RSMs to find the cytokine concentrations that maximized the number of cells in different populations for each 7-day interval. We assumed an ancestor-progeny developmental relationship such that increasing the number of early T-lineage progenitors would have a positive impact on the number of later-stage cells. A desirability function for each population was used to calculate overall desirability, which was maximized using the basin-hopping algorithm (Supplementary Fig. 7)^31^. The optimization objectives were changed throughout the differentiation to reflect the populations present, first maximizing cells in the CD3^-^ populations and shifting to CD3^+^ cells (Fig. 4d).

The top five solutions from the optimization converged to the same overall desirability score indicating that they are global maxima (Fig. 4e and Supplementary Fig. 7). Next, we constructed a three-stage protocol that approximated the 7-day interval optima as closely as possible. We split the assay into the intervals [0, *t*_*1*_], [*t*_*1*_*, t*_*2*_,], and [*t*_*2*_, 42] days, where *t*_*1*_*, t*_*2*_ are multiples of 7 in [7, 42) and *t*_*1*_ < *t*_*2*_. We then averaged the predicted optimal cytokine concentrations within the intervals for every *t*_*1*_*, t*_*2*_ and found the pair that yielded the highest average overall desirability over the entire 42-day assay. Using this approach, we found *t*_*1*_ = 7 and *t*_*2*_ = 21 maintained desirability scores that were close to the 7-day interval scores (Supplementary Fig. 7). The three-stage optimum cytokine concentrations are provided in Supplementary Table 38.

**Fig. 5:**
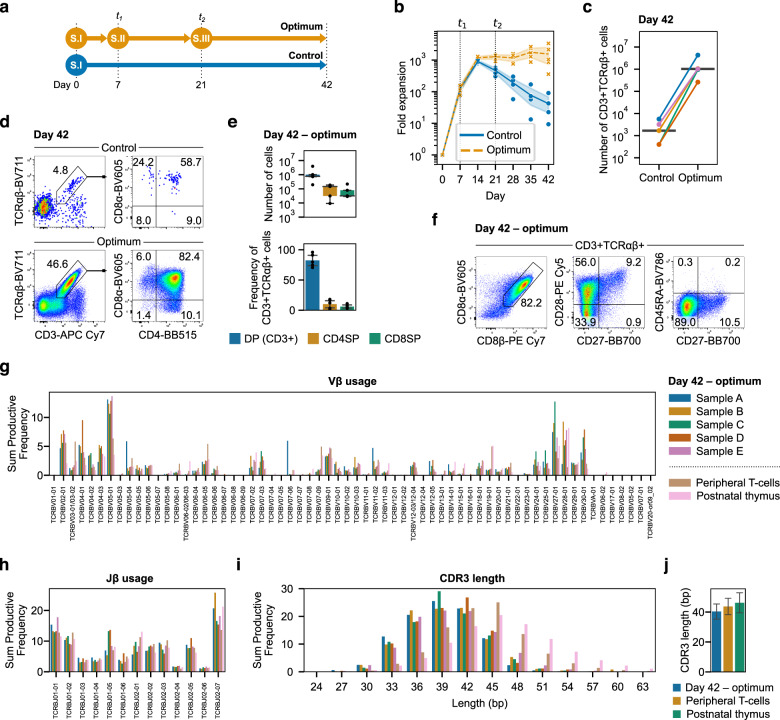
Optimized cytokine concentrations provide stage-specific signals during T-cell development. **a** The three-stage optimum cytokine concentrations were compared to a control that used the first stage concentrations throughout the entire differentiation. All other parameters (seeding density, passage ratio, schedule, etc.) were kept the same for each condition. **b** Fold expansion over 42 days using the optimized three-stage assay. Cytokine concentrations were changed at *t*_*1*_ and *t*_*2*_. Lines represent the mean over time for each condition and the shaded areas are the 95% confidence intervals. **c** Absolute count of CD3^+^TCRαβ^+^ cells on day 42 from 2000 HSPCs seeded per well on day 0. Lines connecting control and optimum are from the same UCB donor and the horizontal line is the median for each condition. **d** On day 42, CD3^+^TCRαβ^+^ cells were predominantly DP with some CD4SP and CD8SP T-cells present. **e** Absolute count and frequency of CD3^+^ DP, CD4SP, and CD8SP T-cells on day 42. The number and frequency of CD4/8 SP cells were comparable at this timepoint. The box plot shows median and interquartile range while the bar plot shows mean ± standard error. **f** On day 42, CD3^+^TCRαβ^+^ cells generated using the optimized cytokines were predominantly CD8αβ^+^ and expressed CD28. A small population of CD28^+^CD27^+^ cells were present and no cells expressed CD45RA. **g** Vβ gene diversity was comparable to peripheral T-cells and postnatal thymocytes. **h** Jβ gene diversity followed similar patterns of recombination to peripheral T-cells and postnatal thymocytes. **i** CDR3 lengths were similar in differentiated T-cells as peripheral T-cells and postnatal thymus. **j** Mean CDR3 length was similar between T-cells differentiated using optimized cytokines, peripheral T-cells, and postnatal thymocytes. From *n* = 5 UCB donors. One donor each of peripheral T-cells and postnatal thymocytes were included for comparison.

We then tested the three-stage system for its ability to support T-lineage development. As a control, we used the first stage cytokine concentrations—similar to our unoptimized concentrations used previously—over the entire 42 days (Fig. 5a). Both the optimum and control conditions yielded comparable numbers of cells during the first 14 days, after which the number of lymphocytes in the control steadily decreased while the optimum plateaued but did not decrease (Fig. 5b). On day 42, the median absolute count of CD3^+^TCRαβ^+^ cells in the optimum was ~600-fold greater than the control: 1.02 M (95% CI: 0.13M–2.91 M) versus 1680 (340–4171) cells from 2000 HPSCs seeded per well at the start of the experiment (Fig. 5c). In both groups, CD3^+^TCRαβ^+^ cells were predominantly DP with a small number of CD4SP (CD4^+^CD8α^−^) and CD8SP (CD4^−^CD8α^+^) T-cells (Fig. 5d, e). We further characterized cells generated with the optimized cytokines. Of the CD3^+^TCRαβ^+^ population, 82.2 ± 3.6% (mean ± standard error) expressed the CD8αβ heterodimer and were transitioning from a CD28^−^CD27^−^ (33.9 ± 6.0%) through CD28^+^CD27^−^ (56.0 ± 6.6%) to CD28^+^CD27^+^ (9.2 ± 1.0%) phenotype (Fig. 5f). CD45RA was not expressed by any CD3^+^TCRαβ^+^ cells. Collectively, this positions the majority of cells as progressing through selection but not yet functionally mature^32^. Bulk VDJ sequencing of the *TRB* locus showed similar patterns of Vβ and Jβ gene usage to peripheral T-cells and postnatal thymocytes (Fig. 5g, h). Likewise, complementarity-determining region 3 (CDR3) lengths were similar between all T-cell sources (Fig. 5i, j). Neither TCR Vα24-Jα18 expressed by invariant NKT-cells nor TCR Vα7.2 expressed by mucosal-associated invariant T (MAIT)-cells were detected by flow cytometry (Supplementary Fig. 8). Thus, the three-stage optimum cytokines enhance survival and/or proliferation to provide a substantial increase in CD3^+^TCRαβ^+^ cells expressing a diverse TCR repertoire. These results validate the optimization methodology used for predicting dynamic cytokine signaling requirements during development.

### Generated conventional T-cells secrete cytokines upon TCR stimulation

**Fig. 6:**
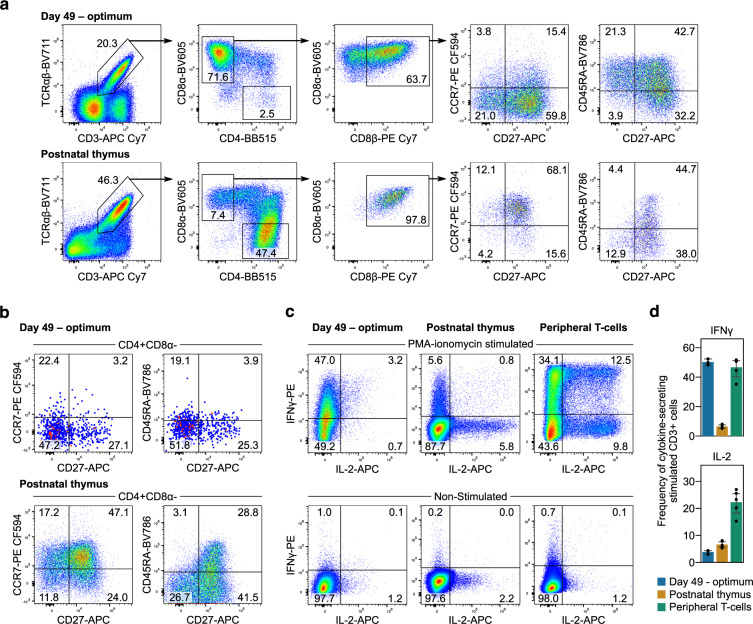
Generation of conventional T-cells that secrete cytokines. **a** Nonspecific TCR stimulation using anti-CD3 monoclonal antibodies with IL-2 administration induced maturation of DP to CD8SP T-cells. These cells predominantly expressed CD8αβ heterodimers and high levels of CD27 and CD45RA. A smaller proportion of CD8SP expressed CCR7 than postnatal thymocytes. **b** A small population of CD4SP T-cells were present and fewer of these cells expressed CD27 and CCR7 than ETN-generated CD8SP or CD4SP postnatal thymocytes. Shown are means from *n* = 3 UCB donors and *n* = 3 postnatal thymus donors. **c** Further stimulation using PMA and ionomycin resulted in secretion of IFNγ with some cells also secreting IL-2. Fewer postnatal thymocytes secreted IFNγ than generated T-cells and none secreted both IFNγ and IL-2. Peripheral T-cells secreted high levels of both IFNγ and IL-2. **d** The frequency of IFNγ secreting cells was comparable between ETN-generated and peripheral T-cells and markedly greater than postnatal thymocytes. The frequency of IL-2 secreting cells was much lower in ETN-generated T-cells and postnatal thymocytes than peripheral T-cells. All cytokine secretion is from viable CD3^+^ cells. **c**, **d** from *n* = 3 UCB donors. One donor each of postnatal thymocytes and peripheral T-cells are shown with *n* = 5 technical replicates.

To measure T-cell function, cells generated using the three-stage ETN system were stimulated for 2 days with an anti-CD3 monoclonal antibody and IL-2 followed by 5 days with IL-2 alone. Of the resultant CD3^+^TCRαβ^+^ cells, 71.6 ± 8.5% were CD8SP T-cells that expressed variable levels of the CD8αβ heterodimer and CD8αα homodimer, consistent with conventional T-cells after TCR stimulation (Fig. 6a)^33^. Compared to the human postnatal thymus, the frequency of CD27 expressing CD3^+^TCRαβ^+^CD8αβ^+^ cells was similar, while fewer ETN-generated cells expressed CCR7. However, the proportion of cells expressing CD45RA was markedly higher than thymocytes. A small population (2.5 ± 2.0%) of CD4SP T-cells was present with fewer expressing CD27 than ETN-generated CD8SP or CD4SP thymocytes (Fig. 6b). Subsequent stimulation with phorbol 12-myristate 13-acetate (PMA) and ionomycin induced IFNγ secretion in 50.2 ± 1.2% and IL-2 secretion in 3.8 ± 0.4% of CD3^+^ cells (Fig. 6c, d). We compared the cytokine secretion of ETN-generated T-cells to postnatal thymocytes and pan-CD3^+^ peripheral T-cells. The thymocytes and peripheral T-cells were first primed using anti-CD3 or anti-CD3/28 with IL-2 and then stimulated using PMA and ionomycin. Few postnatal thymocytes secreted IFNγ (6.5 ± 0.6%) or IL-2 (6.6 ± 0.6%). The frequency of IFNγ secreting peripheral T-cells (46.6 ± 3.1%) was comparable to generated T-cells, although peripheral T-cells secreted much more IL-2 (22.3 ± 2.1%). Thus, cells generated using this technology can mature into phenotypically elaborated CD8SP T-cells that are capable of secreting cytokines upon nonspecific TCR stimulation.

## Discussion

**Fig. 7:**
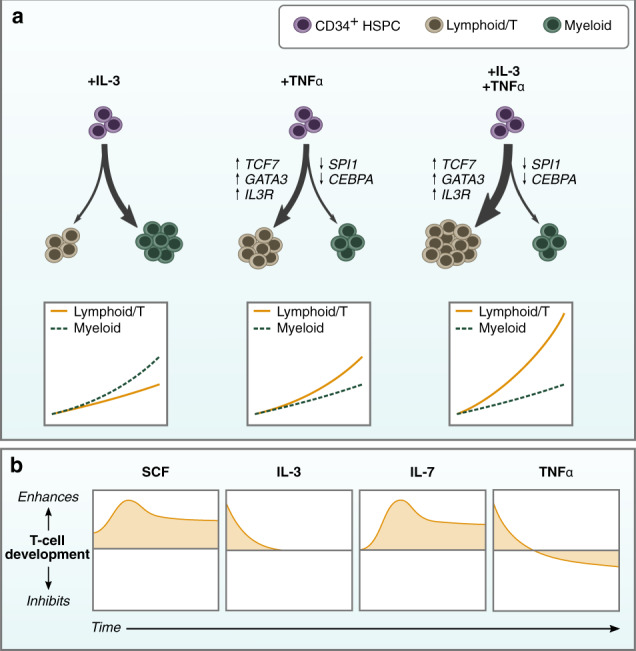
TNFα couples T-lineage differentiation with IL-3-induced proliferation. **a** Individually, IL-3 stimulates proliferation of myeloid-biased CD34^+^ HSPCs while TNFα induces T-lineage differentiation by positively regulating Notch target genes. It also increases the proportion of cells responsive to IL-3 by regulating the IL-3 receptor. When combined, IL-3 provides strong proliferative signals to developing lymphocytes, leading to substantial expansion of T-lineage cells. **b** The positive effects of IL-3 and TNFα are on T-cell specification where cells have limited responsiveness to IL-7. Once T-lineage development is initiated, SCF and IL-7 become the drivers of proliferation, and responsiveness to IL-3 and TNFα is lost. TNFα becomes inhibitory when cells enter developmental stages associated with positive and negative selection.

Current clinically-relevant protocols for generating T-cells from stem cells have, thus far, not been able to generate cells in the quantities or at the purity necessary to make them suitable for translation. Here, we show the benefit of using a defined system for isolating and studying the effects of cytokines on T-cell development and identified interactions between TNFα and the Notch pathway that enhance T-lineage specification. In addition, we found that combining TNFα with IL-3 strongly potentiated proT-cell proliferation independent of effects on myeloid lineage cells (Fig. 7a). We then optimized cytokine concentrations to generate CD3^+^ DP and CD8^+^ T-cells in vitro and to provide an enhanced ETN system, efficiently generating conventional T-cells that express a diverse TCR repertoire.

TNFα was previously reported to accelerate the differentiation of DN1 to the DN2 stage in murine thymocytes, but the mechanism for this effect was not investigated^14^. Our findings show that TNFα has a similar effect on human T-lineage development through the upregulation of Notch target genes *GATA3*, *TCF7*, and *BCL11B*, and the downregulation of *SPI1* and *CEBPA*. The inhibitory effect of TNFα in later stages of T-cell development is likely due to increased apoptosis in cells undergoing selection^34,35^. TNFα is constitutively expressed in the human postnatal thymus, primarily in the cortico-medullary junction (CMJ) and medulla^36^. Thus, we expect progenitors entering the thymus near the CMJ and late DP/SP cells undergoing negative selection in the medulla to experience higher levels of TNFα than those in the cortex. Our findings that TNFα has a positive effect on cells during T-cell specification but a negative effect on later stages of development is consistent with thymus expression.

The combination of IL-3 and TNFα provided a significant enhancement in cell expansion and development to the T-lineage (Fig. 7b). Our results suggest that the signals provided by IL-3 and TNFα are independent: they regulate non-overlapping gene expression that results in a multiplicative increase in proliferation rather than a simple additive effect. The increase in CD123 expression by TNFα likely mediates the proliferative effect, allowing for increased activation by IL-3 of the Janus kinase/signal transducer of and activator of transcription (JAK/STAT) and PI3K/Akt signaling pathways that regulate cell survival and proliferation^37–39^. IL-7 also signals via JAK/STAT and PI3K/Akt, and there is likely overlap in IL-3 and IL-7 induced gene transcription^37^. On its own, IL-3 promoted the early expansion of myeloid lineage cells. TNFα mitigated this effect, likely through its interactions with the Notch pathway. IL-3 is produced by human TECs^40^ and, when combined with SCF, IL-1, IL-6, and IL-7, enables enhanced reconstitution of fetal thymic organ cultures (FTOCs) by mouse proT-cells^12^. Thus, it likely plays a physiological role in human T-lineage development in the thymus. Recently, CD123^+^ thymus seeding progenitors were reported to have the potential to differentiate into dendritic cells^5^. Whether these progenitors also give rise to thymocytes was not clearly resolved. Nevertheless, our work suggests that in the appropriate signaling environment, thymic progenitor cells with T-lineage potential may express CD123 and respond to IL-3.

SCF’s role in proT-cell proliferation and differentiation is well-attributed, but, to our knowledge, its positive effect on the later DP stages is unreported. SCF was shown to promote murine DN1-3 proliferation while inhibiting differentiation to the DP stage in OP9-DL1 cocultures^41^. This may be a result of differences between human and mouse T-cell development, or simply an artifact of the assays in which they were studied (OP9-DL1 versus our defined ETN system). Flt3L had a modest effect on T-cell development. It has been reported to promote survival of bone marrow-resident thymic precursors and stimulate a modest increase in murine proT-cell expansion in FTOCs, but our results suggest it has little effect on human T-cell development in this system and is a candidate for removal^42,43^.

Without stimulation using anti-CD3 antibodies the proportion of CD8SP T-cells by day 42 was relatively low (<10%) and the vast majority of CD3^+^TCRαβ^+^ cells were DP (>80%). Continuing cultures past 42 days would likely increase the number of CD8SPs without requiring nonspecific TCR stimulation that may also expand γδT-cells. Without the provision of a recombined TCR, it is likely that the number of cells may contract slightly as they proceed through negative selection. More CD4SP than CD8SP T-cells were present in cultures on day 42. However, after stimulation, few CD4SPs remained, and most were CD8SP. Small populations of CD4SP T-cells have been reported in OP9-DL1 cocultures, and in vitro differentiated T-cells were shown to express some class-II HLA necessary for positive selection^18,19^. These studies provide conflicting reports about whether CD4SP T-cells generated on OP9-DL1 were functionally mature, as they were deficient for CD27 or CD45RA. In our cultures, CD4SP T-cells expressed less CD27 than CD8SP T-cells at the same timepoint, suggesting that their developmental kinetics are slower or that differentiation conditions need to be optimized for CD4SP maturation.

The CD8SP T-cells generated on day 49 expressed comparable levels of CD27 as postnatal thymocytes but differed in expression patterns of CCR7 and CD45RA. CCR7 plays a role in recruiting thymocytes into the medulla as well as export from the thymus^44^. Low CCR7 expression could indicate that in vitro-generated CD8SP T-cells have yet to pass negative selection. However, the co-expression of CD27 and CD45RA is consistent with post-selection thymocytes and the differences observed could be an in vitro artifact or a result of differences in ontogeny^32^. Longer-term cultures and additional surface markers could be used to resolve these questions in the future. Secretion of IFNγ following stimulation using PMA and ionomycin was comparable between generated T-cells and pan-CD3^+^ peripheral T-cells, although fewer of our generated T-cells secreted IL-2 and were comparable to the postnatal thymus in this respect. This suggests that T-cells generated using our ETN system may be functionally between thymocytes and peripheral T-cells, the latter of which may contain naïve, effector, and memory subsets with different capacities for activation.

Our work demonstrates the utility of building quantitative statistical learning models using a fully defined, serum-free, culture system. As temporal changes in responses to cytokines and growth factors are challenging to study, we split experiments into discrete intervals that could be tested individually and then combined to approximate temporal signaling regimes. Some prior knowledge was necessary to estimate an appropriate length of intervals to capture the developmental dynamics in question. We chose one-week intervals because it was consistent with our existing protocols. However, for periods where development occurs quickly—such as the first week in the ETN system—additional experiments conducted over 3-4 days could augment existing data to iteratively refine model predictions. Likewise, longer experiments that overlap with existing data could be conducted to explore long-term cytokine dependencies. Importantly, this modeling strategy can incorporate new information as it becomes available. For example, if new cytokines or environmental conditions are found to improve the yield or purity of T-cells generated, they can be tested in a similar factorial experiment, added to existing data, and reoptimized. Overall, our statistical learning methodology provides a flexible strategy to predict and optimize time-varying developmental processes.

The application of our ETN system to other stem cell sources, such as induced pluripotent stem cells (iPSCs), is an exciting area of future research. OP9-DL1 cocultures have typically been used to demonstrate T-lineage potential from iPSC-derived HSPCs^45^. Reprogramming of αβT-cell clones into iPSC lines and subsequent differentiation back to the T-lineage has been reported, although these cells may have innate- or γδT-like functional qualities, despite expressing a functional TCRαβ^46,47^. Conventional T-cell development from PSCs has been more challenging, and only recently has it been reported in a serum-free system^48^. Despite observed differences in T-cell potential between umbilical cord blood- and PSC-derived HSPCs^49^, we expect the cytokines identified here to be applicable to T-cell differentiation from iPSCs, although the concentration and timing of administration may need to be adjusted to account for differences in ontogeny.

Future work should explore in vitro T-cell maturation in cells that have been genetically engineered to express a CAR or antigen-specific TCR. While providing a means to control T-cell target specificity, we expect that this would also increase the overall efficiency of T-cells generated in the ETN system. Providing cells with a functional TCR has been shown to block endogenous TCR recombination through allelic exclusion, while providing positive selection signals that prevent death-by-neglect^50,51^. Though there is overlap in TCR/CAR signaling domains, differences in co-stimulatory signals may preclude allelic exclusion in CAR T-cells and are worth exploring further. If this is the case, the CAR could be placed in one *TRAC* allele and the other disrupted using CRISPR/Cas9 to prevent productive TCR transcription. The timing of expression is also important, as an expression of a CAR early in T-cell development can suppress *BCL11B* to induce NK-like differentiation^52^. Nevertheless, the potential to generate CAR or antigen-specific TCR T-cells that do not recombine endogenous TCR loci would reduce the risk of graft-versus-host disease to enable robust and clinically translatable allogeneic T-cell immunotherapies from stem cells.

## Methods

### DL4 production and DL4 + VCAM-1 plate coating

Recombinant human DL4-Fc fusion protein was purchased from Sino Biological or manufactured in-house. The coding sequence of the extracellular domain of human DL4 was cloned upstream of the Fc portion of human IgG1 (including the hinge region) and inserted into a pIRESpuro2 mammalian expression plasmid (Clontech). HEK-293T cells were transfected using CaPO_4_ transfection methods and stably integrated clones were selected using puromycin (2 µg/ml). Secreted DL4-Fc was purified from the supernatant using a HiTrap Protein G affinity column attached to the ATKAprime plus liquid chromatography system (both from GE Healthcare Life Sciences). Tissue culture 96-well plates were coated with DL4-Fc and murine VCAM-1-Fc (R&D Systems) overnight at 4 °C or for 2–4 h at room temperature. To coat, DL4-Fc and VCAM-1-Fc were diluted to 15 and 2.5 µg/ml, respectively, in 50 µl of phosphate-buffered saline (PBS), resulting in a coating concentration of ~24 ng/mm^2^ of DL4-Fc and 4 ng/mm^2^ of VCAM-1-Fc. For experiments with less DL4-Fc, the concentration was adjusted accordingly while maintaining a 50 µl volume of PBS. Wells were washed once with PBS prior to seeding cells to remove unbound protein.

### IMDM + BIT basal medium

Unless otherwise described, cells were cultured in Iscove’s Modified Eagle Medium (IMDM) supplemented with 20% serum substitute (BIT 9500; Stemcell Technologies), 1 µg/ml low-density lipoprotein (Stemcell Technologies), 60 µM ascorbic acid (Sigma), 24 µM 2-mercaptoethanol (Sigma), and 1% penicillin-streptomycin (Invitrogen). This medium was stored at −20 °C. Before use it was thawed, cytokines added, and used within 1 week.

### Human CD34^+^ HSPC enrichment from umbilical cord blood

Umbilical cord blood (UCB) was collected from consenting donors at Mount Sinai Hospital, Toronto, Ontario or BC Children’s Hospital, Vancouver, British Columbia, in accordance with institutional research ethics board policies. Mononuclear cells were isolated via density gradient centrifugation using Lymphoprep (Stemcell Technologies). CD34^+^ cells were isolated using the EasySep Human CD34^+^ Positive Selection Kit (Stemcell Technologies) to >90% purity according to the manufacturer’s instructions (Supplementary Fig. 1). Isolated CD34^+^ HSPCs were cryopreserved in FBS with 10% DMSO and stored in vapor phase nitrogen. Prior to use, cells were thawed in a 37 °C water bath and 10x volume of 37 °C IMDM + BIT was added dropwise. Cells were then centrifuged for 7 min at 300×*g* and resuspended in IMDM + BIT with the appropriate cytokines as described. For certain experiments, enriched CD34^+^ cells were sorted into CD34^+^CD38^lo/−^ and CD34^+^CD38^+^ fractions using the FACS Aria cytometer (Beckman Coulter).

### Human postnatal thymocytes enrichment

Human thymus was collected from patients 3 years or younger while undergoing cardiovascular surgery after parental consent at BC Children’s Hospital, Vancouver, British Columbia, in accordance with institutional research ethics board policies. Thymus tissues were washed with RPMI 1640 (ThermoFisher) with 100 µg/ml DNase I (Roche), thinly sliced with a scalpel, and then dissociated using gentleMACs Dissociator (Miltenyi Biotec). Isolated thymocytes were washed once with Hanks Balanced Salt Solution (HBSS) with 2% FBS (Gibco; collectively referred to as HF buffer) and then incubated with ACK lysing buffer (Lonza) for 5 min at room temperature. Thymocytes were rinsed again with HF buffer and passed through a 40 µM filter to create a single-cell suspension. Isolated thymocytes were subsequently cryopreserved in FBS with 10% DMSO and stored in vapor phase nitrogen. Prior to use, thymocytes were thawed in a 37 °C water bath before adding 10x volume of 37 °C HBSS + 10% FBS dropwise. Thymocytes were then centrifuged for 10 min at 300×*g* and stained for flow cytometry or resuspended in IMDM + 10% Hyclone FBS (Cytiva) with stage-III optimized cytokines at 1–2 M cells/ml and cultured for 48 h on DL4 + VCAM-1 coated plates prior to nonspecific TCR stimulation and cytokine secretion experiments.

### Human peripheral T-cells

Pan-CD3^+^ peripheral T-cells were purchased from HemaCare (PB03C-1). Cells were thawed in a 37 °C water bath before adding 10x volume of 37 °C HBSS + 10% FBS dropwise. T-cells were then centrifuged for 10 min at 300×*g* and resuspended in 37 °C IMDM + 10% Hyclone FBS (Cytiva) with 10 ng/ml IL-2 (R&D Systems) at 1–2 M cells/ml and cultured for 48 h prior to nonspecific TCR stimulation and cytokine secretion experiments.

### HSPC culture on DL4 + VCAM-1

For experiments 7 days or longer, HSPCs were seeded at 1000–4000 cells/well of a 96-well plate on DL4 + VCAM-1-coated surfaces. Experiments shorter than 7 days (CFSE and qPCR) were seeded at 15,000–25,000 cells/well to provide enough cells for analysis. Cells were cultured in 100–200 µl of IMDM + BIT. The cytokines used as a control condition for cytokine screening experiments were SCF, Flt3L, TPO, and IL-7 (all from R&D Systems) at 100 ng/ml (Fig. 1) and 20 ng/ml afterward, unless otherwise mentioned. All other cytokines were purchased from R&D and used as described. For Notch pathway inhibition experiments (Fig. 2), the γ-secretase inhibitor (2 S)-*N*-[(3,5-Difluorophenyl)acetyl]-l-alanyl-2-phenyl]glycine 1,1-dimethylethyl ester (DAPT; R&D Systems) or dimethyl sulfoxide (DMSO; Sigma) was added to media at the concentrations indicated.

### Flow cytometry analysis of surface markers and intracellular cytokines

Adherent cells were collected from DL4 + VCAM-1 surfaces using vigorous pipetting or enzymatically dissociated using TrypLE Express (ThermoFisher). Samples from Figs. 1–3 were stained and collected with an LSRFortessa cytometer (BD Biosciences). Collected cells were rinsed once with HF buffer containing Fc block (BD Biosciences) then stained with fluorophore-conjugated antibodies diluted in HF buffer for 15 min on ice. Cells were rinsed with HF buffer to remove unbound antibodies and resuspended in HF buffer with 7-AAD viability dye (1:1000 dilution; Life Technologies) for analysis. Compensation and gating was with FlowJo X software. The antibodies used in these experiments are listed in Supplementary Table 39. Samples in Figs. 4–6 were collected with a CytoFLEX LX cytometer (Beckman Coulter). Collected cells were rinsed with PBS with Fc block and stained with Zombie-UV viability dye (BioLegend) for 15 min at room temperature. Cells were then stained with fluorophore-conjugated antibodies diluted in Brilliant Stain Buffer (BD Biosciences). Cells were rinsed once with HF buffer to remove unbound antibodies and resuspended in HF buffer for analysis. The antibodies used in these experiments are listed in Supplementary Tables 40, 41. For intracellular cytokine secretion, cells were stained with Zombie-UV like above then fixed and permeabilized using the Cytofix/Cytoperm kit (BD Biosciences) according to the manufacturer’s instructions. The antibodies used in these experiments are listed in Supplementary Table 42. Compensation was performed with the cytometer software (CytExpert v2.3) and gating was performed with FlowJo X. Further analysis and statistics was with the R (version 3.3.2) or Python (version 3.7) programming languages.

### Proliferation assays

Cells were stained with 2.5 µM CellTrace CFSE (ThermoFisher) in PBS and incubated at 37 °C for 8 min. CFSE was quenched by adding five times the volume of IMDM + BIT medium and incubating at 37 °C for 5 min. Cells were then resuspended in fresh media and seeded on DL4 + VCAM-1 for culture. For analysis, cells were collected and analyzed using an LSRFortessa cytometer. Proliferation was modeled using FlowJo X software and further analysis was performed with R.

### Nonspecific TCR stimulation

ETN-generated T-cells (on day 42) were seeded at 1–2 M cells/ml in plates coated with DL4 + VCAM-1 and 500 ng/ml anti-CD3 monoclonal antibody (OKT3 clone; Biolegend) and cultured for 2 days in IMDM + BIT with stage-III optimized cytokines plus 10 ng/ml IL-2 (R&D Systems). Cells were then passaged to new DL4 + VCAM-1-coated plates (without anti-CD3) in fresh medium and cultured for 5 additional days. Postnatal thymocytes were similarly stimulated with anti-CD3 plus DL4 + VCAM-1 except IMDM + BIT was replaced with IMDM + 10% Hyclone FBS (Cytiva) basal medium with the same cytokines. Peripheral T-cells were stimulated on the same schedule but without DL4 + VCAM-1 and with 1 µg/ml anti-CD3 and 3 µg/ml anti-CD28 (CD28.2 clone; Biolegend) for the first two days in IMDM + 10% Hyclone FBS with 10 ng/ml IL-2.

### Cytokine secretion

Cells were seeded at 50,000–100,000 cells/well of a 96-well plate in 200 µl of 37 °C IMDM + BIT medium containing 25 ng/ml phorbol 12-myristate 13-acetate and 1 µg/ml ionomycin (both from Sigma). After 1 h, 3 µg/ml of brefeldin A was added and cells were cultured for an additional 5 h. Cells were fixed and stained for flow cytometry as described above.

### Quantitative PCR

Cells were lysed and RNA isolated using the PureLink RNA Micro Kit (Invitrogen) according to the manufacturer’s protocol. cDNA was reverse transcribed from RNA using SuperScript III Reverse Transcriptase (Invitrogen) according to the manufacturer’s protocol. cDNA was amplified with primers and FastStart SYBR Green Mastermix (Roche) using QuantStudio 6 Flex (Applied Biosystems). Relative expression of individual genes was calculated by the delta cycle threshold (ΔCt) method and normalized to β-actin. PCR primer sequences are available in Supplementary Table 43.

### VDJ repertoire sequencing

Genomic DNA was extracted from cells using QuickExtract DNA Extraction Solution (Lucigen) and diluted using Tris-EDTA buffer. Library preparation and sequencing of the *TRB* gene locus was by Adaptive Biotechnologies using the ImmunoSEQ survey resolution assay^53^. Data visualization was with Python (version 3.7). Sequence data for a peripheral T-cell population used for comparison was provided by Adaptive Biotechnologies and is publicly available on immuneACCESS (Subject_19; http://adaptivebiotech.com/pub/tcrbv4-control).

### Cytokine screens

For screening experiments, a Definitive Screening Design (DSD)^54^ was constructed with JMP14 software where each cytokine was tested at three concentrations. Cytokine concentrations for initial screening experiments are shown in Supplementary Table 1 and for follow-up experiments in Supplementary Table 8. Absolute cell numbers were acquired for each cell population using flow cytometry and, for initial experiments, a *z*-score was calculated relative to a control condition to gain a better- or worse-than estimate. The control condition was the cytokines used previously in the DL4 + VCAM-1 assay as in Shukla et al.^22^. For follow-up experiments, the regression model was fit to the cell counts instead of *z*-scores. Stepwise regression using the minimum Bayesian information criterion was calculated using JMP14 software. Regression coefficients and statistics are provided in Supplementary Tables 2–14. Additional information about screening experimental design and analysis can be found in Supplementary Methods.

### Response surface methodology (RSM) models of cytokine dose responses

RSM experiments were orthogonal central composite designs (CCD) comprising six cytokines at five concentrations designed with JMP14 software (Supplementary Table 15). Throughout the experiment, cells were passaged every 7 days onto freshly DL4 + VCAM-1 coated plates in 100 µl of fresh media. An additional 100 µl of media was added mid-week between passages. Absolute cell counts were acquired for all populations of interest using flow cytometry and multivariate least-squares regression with JMP14 software was used to construct the RSM models. Regression coefficients and statistics are provided in Supplementary Tables 16–37. Additional information about RSM experimental design and analysis can be found in Supplementary Methods.

### Optimizing RSM models for T-cell maturation

Regression coefficient estimates were used to build models in custom Python code (version 3.7). Desirability functions were used to maximize multiple RSM models simultaneously using the Basin-Hopping algorithm in the *SciPy* library^31,55^. Additional information about the optimization procedure can be found in Supplementary Methods.

### Statistical analysis

Except for screening and RSM experiments, all statistics were calculated in R (version 3.3.2). A Shapiro–Wilks normality test was used to determine whether data could be appropriately modeled by a Gaussian distribution. If data was non-Gaussian (Shapiro–Wilks *p* < 0.05), a nonparametric Kruskal–Wallis test with Dunn’s post hoc analysis was used, and the false discovery rate was minimized using the Benjamini–Hochberg *p* value adjustment. Otherwise, one-way ANOVA with Tukey post hoc analysis was employed.

### Reporting Summary

Further information on research design is available in the Nature Research Reporting Summary linked to this article.

## Supplementary information

Supplemental Material

REPORTING SUMMARY

## Data Availability

Regression coefficient estimates for DSD and RSM experiments are provided in the supplemental materials provided with this article. *TRBV* and *TRBJ* gene usage data are available via immuneACCESS (DOI: 10.21417/JME2021NPJRM) and raw *TRB* gene sequencing data is available from Gene Expression Omnibus (GSE191086). For other original data, please contact peter.zandstra@ubc.ca.

## Code availability

The custom Python code used to build and optimize RSM models from coefficient estimates is available at gitlab.com/stemcellbioengineering/polynomialfeatures (v1.1).
