# Supplementary material for: Multi-objective optimization reveals time- and dose-dependent inflammatory cytokine-mediated regulation of human stem cell derived T-cell development
Source: NPJ Regen Med. 2022 Jan 27;7:11. doi: 10.1038/s41536-022-00210-1 (PMC8795204; doi:10.1038/s41536-022-00210-1)
Supplement: Supplementary file 1 — Supplemental Material [file 41536_2022_210_MOESM1_ESM.pdf]

SUPPLEMENTARY METHODS

Definitive Screening Design (DSD) Experiments

DSD are a method to screen a large number of factors in a relatively small experiment size[1]. They are three-level designs of at least $2k + 1$ runs, where k is the number of factors being tested and levels are coded as -1,0,1. The design comprises k fold-over pairs, where one factor is set at the mid-range (0) and all other factors are set to the extrema (-1 or 1). An added centre run with factors set to 0 enables DSDs to estimate all quadratic effects X_{ii}^2. In a DSD, all main effects X_i are independent of any two-factor interactions X_iX_j and no two-factor interactions are confounded with each other, although they may be correlated. For designs of 6 or more factors, it is possible to fit a full quadratic model to any 3 factors. DSDs may also include orthogonal blocks, allowing variance due to biological replicates to not confound factor estimates[2]. Experimental data is fitted with a quadratic model to estimate responses to screened factors.

We designed a 15-factor DSD with blocking to screen for cytokines that induce proliferation during T-cell specification of $CD34^+$ HSPCs on DL4+VCAM-1 (Supplemental Table 1). Two sequential DSD experiments were performed. The first, from day 0-7 and the second from day 7-14. In the latter, cells were cultured in the mid-range (0) concentrations for the first 7 days then passaged at equal densities to the test conditions. The motivation for this was to separate effects on early hematopoietic progenitor cells from proT-cells. Thus, it would allow us to identify if a factor had a positive effect in one stage and a negative effect in the other. As a control, we included a condition using the cytokines SCF, Flt3L, TPO, and IL-7 at the concentrations used in Shukla *et al*[3]. To get a better- or worse-than estimate of cytokine effects relative to the control, a z-score was calculated for each test condition:

$$z = \frac{test - control}{SE(control)}$$

where $SE(\cdot)$ is the standard error. At the end of each experiment, the number of total cells, $CD7^+$ lymphoid cells, and $CD7^+CD5^+$ proT-cells was measured using flow cytometry. Absolute cell counts were square root or log transformed as necessary to satisfy model assumptions that the residuals be approximately normally distributed. Due to the small size of DSD experiments, they rely on only a few factors being active. To avoid user bias in determining which factors to include, stepwise regression was used to automatically select the best model for the data. The minimum Bayesian information criterion (BIC) was used as the stopping rule during the procedure. BIC seeks to minimize the number of model parameters while maximizing the likelihood function, thus selecting the smallest model with the best fit possible. The value of the β coefficient estimates for the resulting models were ranked by magnitude and visualized in order to determine which cytokines elicited the strongest effects on the populations measured. All parameter estimates are provided in Supplemental Tables 2-7.

We also constructed a 10-factor DSD as a follow-up experiment to confirm our results and to estimate working concentrations for IL-3 and TNFα. The same procedure was applied except that the relative cell counts were used instead of the z-score. The cytokines used and their concentrations are provided in Supplemental Table 8 while the parameter estimates are provided in Supplemental Tables 9-14.

Response Surface Methodology (RSM) Models of Cytokine Dose Responses

Our objective in using RSM was to find a quadratic approximation of the non-linear dose response of developing T-cells to cytokines. Recognizing that responsiveness to cytokines may change as cells differentiate, we designed successive 7 day experiments where cells were cultured on DL4+VCAM-1 with different concentrations of test cytokines. At the end of 7 days we measured the absolute cell count for each population of interest using flow cytometry. We repeated this until day 42 for a total of six 7-day intervals. For each interval after day 0-7, a pool of cells were cultured on DL4+VCAM-1. When beginning an RSM experiment these cells were passaged at equal density to the test conditions in order to measure the relative growth expansion for each condition. Three populations were measured in the first 14 days: proT ($CD7^{+}CD5^{+}$), CD4ISP ($CD4^{+}CD8a^{-}CD3^{-}$), and early DP cells ($CD4^{+}CD8a^{+}CD3^{-}$). From day 14 onward we looked at early DP, late DP ($CD4^{+}CD8a^{+}CD3^{+}$) and CD8SP ($CD4^{-}CD8a^{+}CD3^{+}$) cells. A small number of CD4SP ($CD4^{+}CD8a^{-}CD3^{+}$) cells were present at later timepoints but were infrequent and were thus excluded. All populations were mutually exclusive except for proT-cells which could potentially overlap with any of the other populations. However, our experience is that most cells become $CD7^{low/-}$ as they start to express CD4. Therefore, the proT-cell population does not completely overlap with any of the others.

In order to fit a quadratic function to experimental data, a 5-level, 54 condition central composite design (CCD) for 6 factors with orthogonal blocking was constructed from a 2^{6-1} fractional factorial experiment (32 conditions) with added centre (10 conditions) and axial points (12 conditions). The concentrations of each cytokine were coded: (-1, 1) for the fractional portion, 0 for the centre points, and α=2.366 for the axial points. Axial points are where one cytokine is set to $\pm\alpha$ while all other cytokines are set to 0. Combined with the centre points where all cytokines are set to 0, they enable the estimation of quadratic dose responses. Scaled levels could be converted to cytokine concentrations by $c = c_0 * 3.5^X$, where X is the scaled level and c_0 is the concentration when $X = 0$. This ensured that concentrations were continuous so that interpolated levels could be converted to physical concentrations. The value of c_0 for each cytokine was set to ensure that the working concentrations used in previous experiments were within range (Supplemental Table 15). The exponent base (3.5) was chosen to ensure that the range of cytokines was wide enough so that the change in response would be greater than measurement noise or biological variation.

A quadratic model was fit to each population for each time interval that has the form:

$$\hat{Y} = \beta_0 + \sum_{i=1}^{k} \beta_i X_i + \sum_{i=1}^{k-1} \sum_{j=i+1}^{k} \beta_{ij} X_i X_j + \sum_{i=1}^{k} \beta_{ii} X_{ii}^2 + \epsilon$$

where $\hat{Y}$ is the response variable (number of cells), X are the coded factor levels, and β, ϵ are the parameters to be estimated using regression. The X_i terms are main effects, $X_i X_j$ are two-factor interactions where the effect is greater than additive, and X_{ii}^2 are quadratic terms that result in a non-linear response. Accurately predicting model parameters assumes that residuals be approximately normally distributed. To ensure this, we transformed the absolute cell counts using a square root transform $\hat{Y} = \sqrt{Y}$. After fitting, the residual distributions for the models were fit with a normal distribution and a Shapiro-Wilks Goodness-of-Fit test was applied to the fitted distribution. A test value of $p>0.05$ indicated that the residuals were approximately normal.

Least-squares regression was used to estimate model parameters. For each time interval a multiple response model was used that fit the same parameters to all responses (cell populations). A full model was fit and then reduced by removing terms that were not significant to p<0.01, unless the non-significant term was part of a higher order effect. Plots of actual versus predicted values were used to check for potential outliers. In some cases, an axial condition for SCF and IL-7 was a notable outlier. In these instances, a new model was built that included a third order term (X^3) for that factor which improved the overall fit. While the model terms were the same for all cell populations within a given time interval, models from different time intervals could use different terms. All parameter estimates are provided in Supplemental Tables 16-36.

Optimization of RSM Models per 7 Day Interval

The utility of regression models created using RSM is their ability to interpolate between test conditions to predict optimal responses. We were interested in optimizing these models to predict the cytokine concentrations that are best suited to T-cell maturation in the DL4+VCAM-1 assay. We assumed an ancestor-progeny relationship (proT → CD4ISP → early DP → late DP → CD8SP) where increasing the number of ancestor cells at time t_n increases the number of progeny at time t_{n+1}. Therefore, if we are interested in producing as many late DP or CD8SPs as possible, we first want to find conditions that increase proT, then CD4ISP, then early DP numbers. However, differentiation is not synchronous and any of these populations may be present in cultures at the same time. This requires a multi-objective optimization strategy to find cytokine concentrations that best suit the growth and development of multiple cell types at once.

We used desirability functions to optimize multiple objectives simultaneously[4]. For each RSM model, we defined a piecewise objective function to maximize the response $\hat{Y}_i$:

$$d_i(\hat{Y}_i) = \begin{cases} 0 & \hat{Y}_i < L_i \\ \left(\dfrac{\hat{Y}_i - L_i}{U_i - L_i}\right)^s & L_i \le \hat{Y}_i \le U_i \\ 1 & \hat{Y}_i > U_i \end{cases}$$

where U_i, L_i are upper and lower limits, respectively, that set the boundaries of the objective. This function scales the response to [0,1] where responses $\hat{Y}_i < L_i$ are undesirable and $\hat{Y}_i > U_i$ are most desirable. We set $L_i = 0$ and $U_i = 2\hat{Y}_{i,max}$, where $\hat{Y}_{i,max}$ is the maximum value when sweeping one factor at a time from low to high while holding the others at 0. When the scaling factor $s = 1$ the function is linear for $L_i \le \hat{Y}_i \le U_i$.

The overall desirability combines all individual desirability functions using the geometric mean:

$$D = \left(\prod_{i=1}^{p} d_i\right)^{\frac{1}{p}}$$

for $i = 1,2, \dots, p$ objectives. Notice that for any $d_i(\hat{Y}_i) = 0$ the overall desirability is 0. The utility of this approach is that by combining multiple objectives into one single objective we can use any single objective optimization algorithm. We used the Basin-Hopping algorithm from the *SciPy* library in Python 3.7 (`scipy.optimize.basinhopping`) which is well-suited to

multivariable multimodal optimization problems[5]. Beginning from a random initialization X_0, the algorithm cycles through the following steps:

1. Perform a random perturbation in X.
2. Local minimization using the Nelder-Mead simplex method.
3. Accept or reject the new X based on the minimized function value $(-D)$.

Because the algorithm is a stochastic global optimizer, there is no way to know whether the solution returned is the true global minimum. Therefore, this procedure was repeated from 25 random initializations and the best 5 solutions were retained. To ensure that solutions were within the concentration levels tested, we constrained the search space using the $L2$-norm of X. By setting $D = 0$ when $\|X\|_2 > 2.366$, all solutions outside of the hypersphere design space were undesirable and therefore excluded. This prevented extrapolation beyond the concentrations tested where prediction error increases.

Each time interval was optimized separately using the above procedure. This yielded an optimal set of cytokine concentrations for each interval. Provided the optimizer converged on the global minimum, the overall desirability for the best 5 solutions are equal. However, this does not mean that the predicted cytokine concentrations will be the same. This is to be expected from and is typical for multi-objective optimization. These predicted cytokine concentrations comprise samples from the Pareto front – optimum solutions where one individual objective cannot be improved further without detrimentally affecting another objective. This is useful information for us because objectives with a larger variance in predicted optima indicate cytokines whose concentration is not as crucial as one with a small variance. Therefore, this gives us a method to qualitatively assess the importance of different cytokines at each interval in the differentiation.

Optimizing Averaged Cytokine Concentrations Over Time

The predicted optimum cytokine concentrations for each interval are accurate for the RSM models used in the optimization (provided the global minimum is found). However, the RSM models are statistical and their fit may be impacted by technical noise or measurement error that may result in fluctuations in predictions that are not biologically significant. Additionally, changing cytokine concentrations every 7 days is cumbersome to implement in the laboratory. To address both of these issues, we split the assay into three intervals and selected the cytokine concentrations to use by averaging the 7-day optima within each interval. Rather than split the assay into three 14 day intervals, we opted to find those which would provide cytokine concentrations that were as close to the 7-day optima as possible. To do this, we split the assay into the intervals [0, t_1], [t_1, t_2,], and [t_2, 42] days, where t_1, t_2 are multiples of 7 in [7,42) and $t_1 < t_2$. For every pair of t_1, t_2 we averaged the optimal cytokine concentrations between the intervals and then calculated the overall desirability score for each 7-day interval using the averaged concentrations. This allows us to change cytokines concentrations as often as every 7 days during periods of rapid differentiation (ie. T-lineage specification) while periods of slower differentiation may use the same cytokine concentrations for 14 days or longer. Averaging over multiple time intervals also provides a consensus of optima to smooth out fluctuations due to noise.

Supplementary References

1. Jones, B. & Nachtsheim, C. J. A Class of Three-Level Designs for Definitive Screening in the Presence of Second-Order Effects. *Journal of Quality Technology* **43**, 1–15 (2011).
2. Jones, B. & Nachtsheim, C. J. Blocking Schemes for Definitive Screening Designs. *Technometrics* **58**, 74–83 (2016).
3. Shukla, S. *et al.* Progenitor T-cell differentiation from hematopoietic stem cells using Delta-like-4 and VCAM-1. *Nat Methods* **14**, 531–538 (2017).
4. Derringer, G. & Suich, R. Simultaneous Optimization of Several Response Variables. *Journal of Quality Technology* **12**, 214–219 (1980).
5. Wales, D. J. & Doye, J. P. K. Global Optimization by Basin-Hopping and the Lowest Energy Structures of Lennard-Jones Clusters Containing up to 110 Atoms. *J. Phys. Chem. A* **101**, 5111–5116 (1997).

SUPPLEMENTARY FIGURES

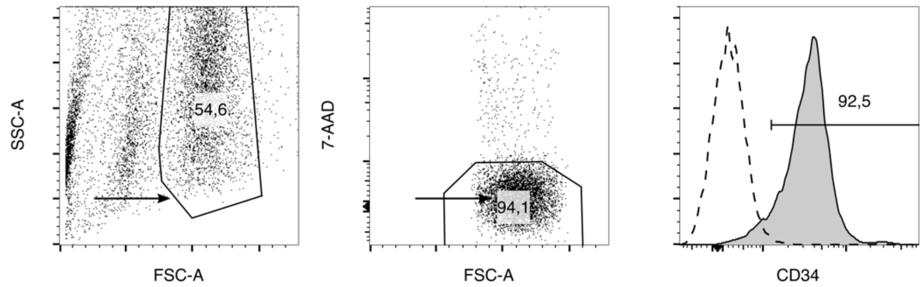

Supplementary Figure 1. CD34⁺ HSPC enrichment. Representative flow cytometry plot showing purity of umbilical cord blood-derived CD34+ HSPCs after isolation using EasySep magnetic beads.

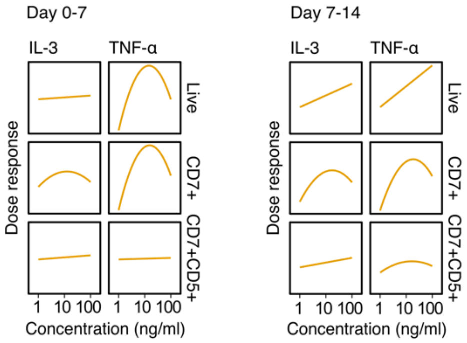

Supplementary Figure 2. IL-3 and TNFα dose response. Top cytokines from the initial screening experiment were used for second validation DSD experiment at a different range of concentrations. This was used to set the working concentration for IL-3 and TNFα to 10 and 5ng/ml, respectively. From n=2 independent UCB donors.

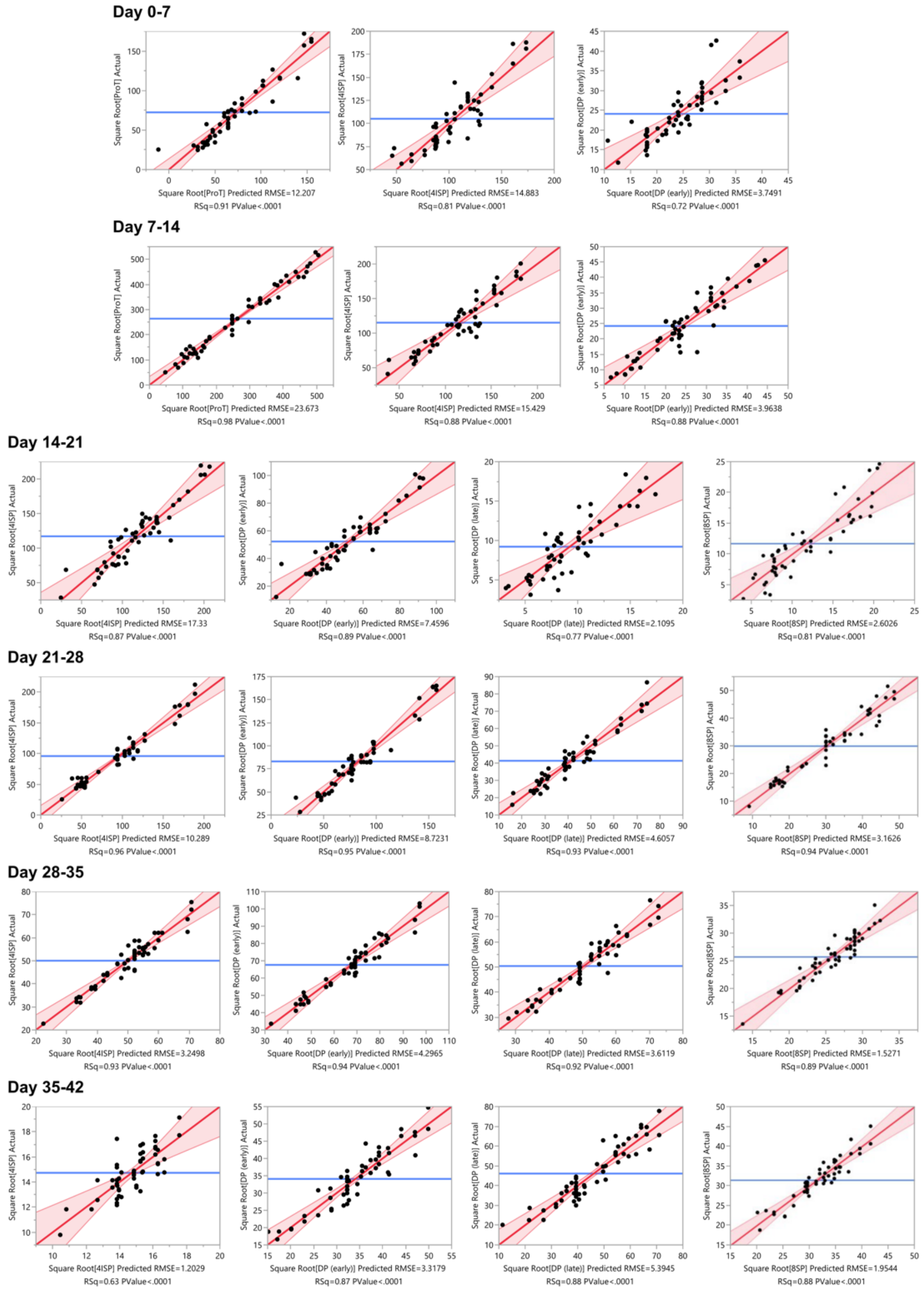

Supplementary Figure 3. Fit of RSM regression models. Actual vs. predicted plots for RSM models. These were used to check for outliers and assess the quality of the model fit. DP (early) are $CD3^{-}$ and DP (late) are $CD3^{+}$.

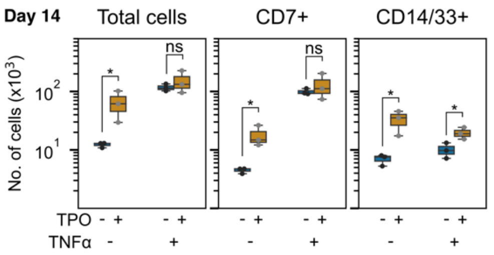

Supplementary Figure 4. Differentiation with and without TPO. Removing TPO from cultures containing TNFα reduces CD14/33$^+$ myeloid cell numbers without affecting total or CD7$^+$ cell numbers. *$p<0.05$ for n=3 independent UCB donors.

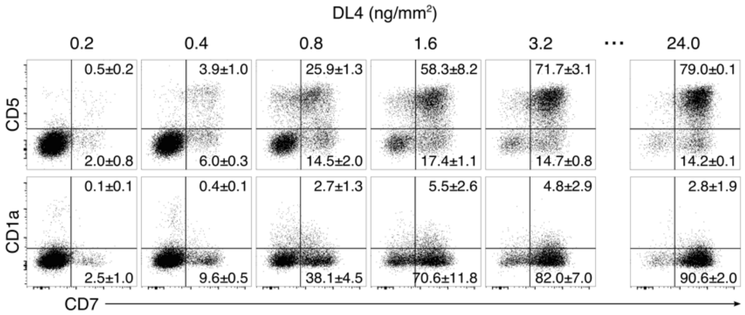

Supplementary Figure 5. Titrating DL4 coating concentration. IL-3+TNFα support T-lineage development with a 7.5-fold reduction in DL4 coating concentration. From n=2 independent UCB donors.

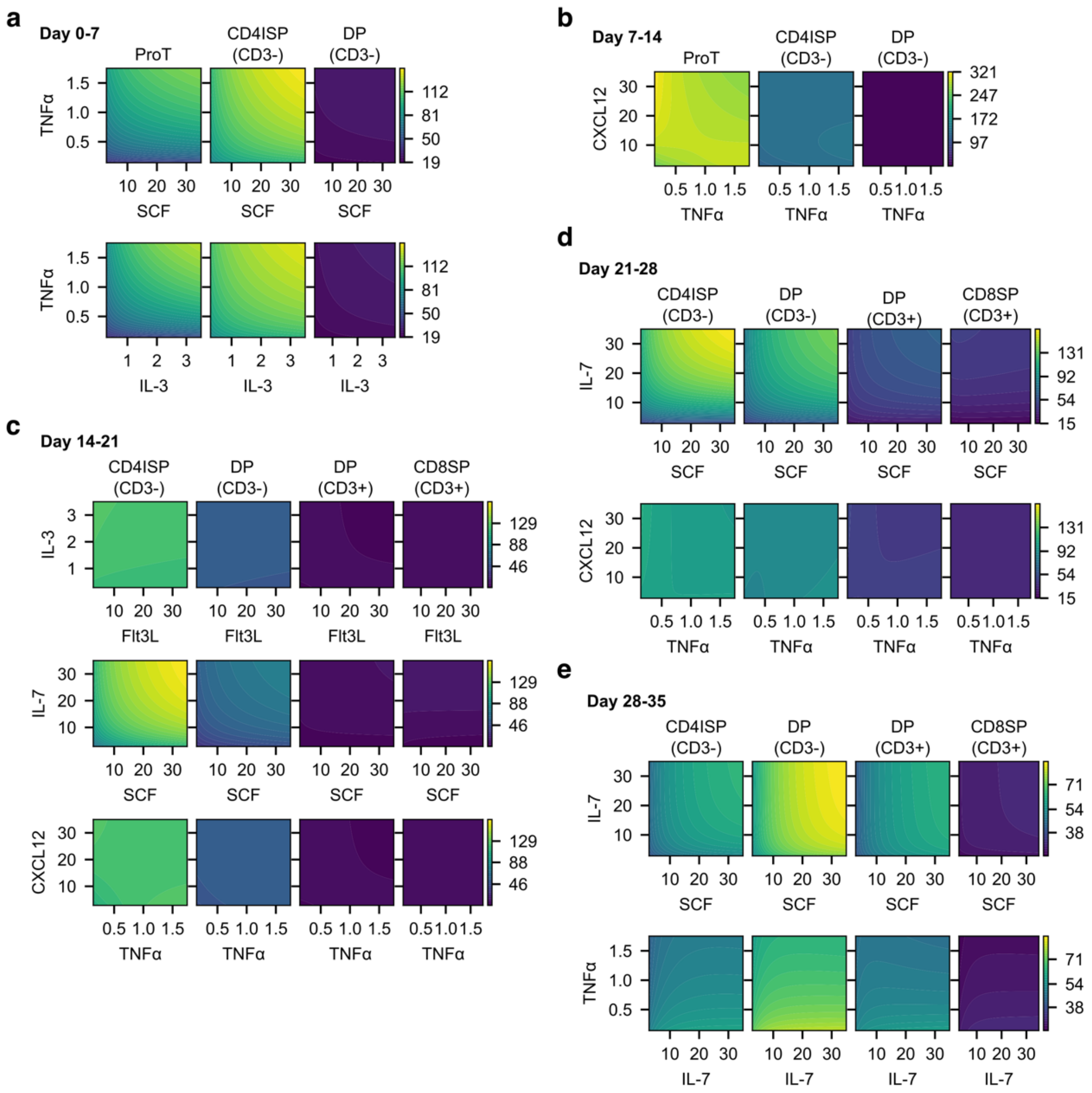

Supplementary Figure 6. Predicted two factor interactions in RSM models. Two factor interactions (2FIs) between cytokines for each 7-day interval. The *x* and *y* axis are the cytokine concentration (ng/ml) while the colorbar shows square root cell counts. (a-b) 2FIs for proT, CD4ISP, and early DP cells from day 0-14. (c-e) 2FIs for CD4ISP, early and late DP, and CD8ISP cells from day 14-35. No 2FIs were detected during day 35-42. RSM was constructed using n=3 pooled UCB donors

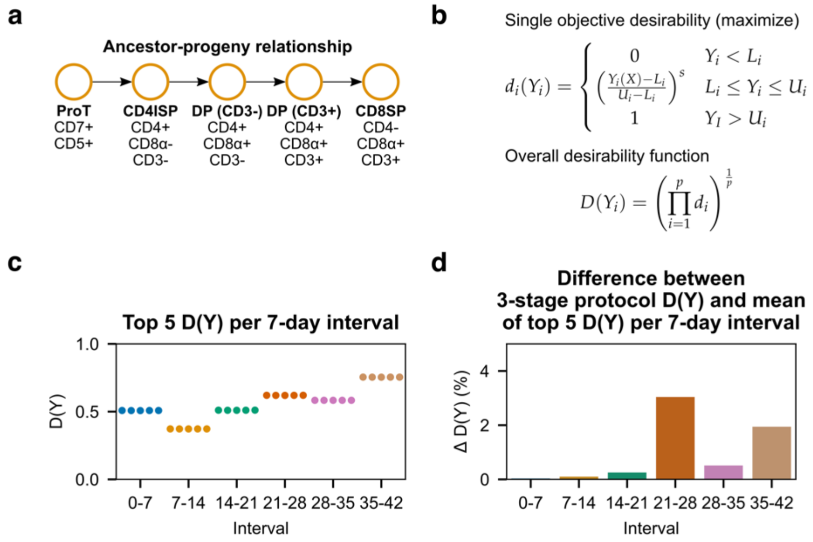

$$d_i(Y_i) = \begin{cases} 0 & Y_i < L_i \\ \left(\frac{Y_i(X) - L_i}{U_i - L_i}\right)^s & L_i \le Y_i \le U_i \\ 1 & Y_I > U_i \end{cases}$$

$$D(Y_i) = \left(\prod_{i=1}^{p} d_i\right)^{\frac{1}{p}}$$

Supplementary Figure 7. Optimizing stage-specific cytokine concentrations. (a) The assumed developmental relationship between proT-cells and more mature phenotypes and the surface markers used to define those subsets. (b) A single objective desirability function scales values of Y_i between (0,1). The lower (L_i) and upper (U_i) set points provide a range for scaling and values that fall outside are set to 0 or 1, respectively. The overall desirability is calculated as the geometric mean of multiple single desirability functions. (c) The top 5 of 25 overall desirability scores were all equally desirable. (d) The overall desirability for each 7-day interval was calculated using the cytokine concentrations from the 3-stage design and compared to the mean of the top 5 desirability scores for each 7-day interval. The maximum difference was 3.0% for day 21-28 while most differences were <1%.

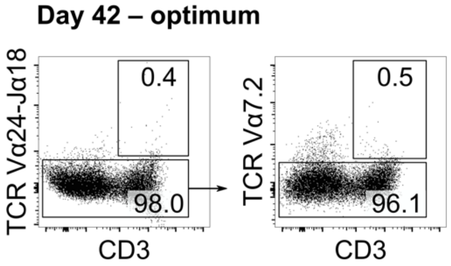

Supplementary Figure 8. Invariant TCR expression in T-cells generated using optimized cytokines. TCR Vα24-Jα18 expressed by invariant NKT-cells or TCR Vα7.2 expressed by mucosal-associated invariant T (MAIT)-cells were not detected in T-cells at day 42 of differentiation.

SUPPLEMENTARY TABLES

Supplementary Table 1 (Related to Figure 1). Factor concentrations for screening experiments.

	Scaled factor level			
**Factor**	**-1**	**0**	**1**	
SCF	1.5	15	150	ng/ml
Flt3L	1.5	15	150	ng/ml
TPO	1.5	15	150	ng/ml
IL-1a	0.01	0.1	1	ng/ml
IL-1b	0.01	0.1	1	ng/ml
IL-3	0.25	2.5	25	ng/ml
IL-6	0.25	2.5	25	ng/ml
IL-7	1.5	15	150	ng/ml
IL-15	0.25	2.5	25	ng/ml
IL-18	0.25	2.5	25	ng/ml
IL-21	0.25	2.5	25	ng/ml
IFNy	0.25	2.5	25	ng/ml
TNFa	0.25	2.5	25	ng/ml
RANTES	0.25	2.5	25	ng/ml
CXCL12	0.25	2.5	25	ng/ml

Supplementary Table 2 (Related to Figure 1). Linear regression model terms for total cells from day 0-7. Provided as raw values at the default precision in JMP 13 software that was used for regression.

Term	Scaled Estimate	Std Error	t Ratio	Prob>|t|
Intercept	78.5548504	4.15784548	18.8931625	1.17E-14
SCF	31.3582336	1.20371042	26.0513102	1.79E-17
Flt3L	2.34525045	1.20371042	1.94835104	0.06486147
IL-1a	2.54866038	1.20371042	2.11733681	0.04633552
IL-1b	1.112316	1.20371042	0.92407275	0.36594547
IL-3	21.1497617	1.20371042	17.5704732	4.91E-14
IL-7	2.96704437	1.20371042	2.46491541	0.02241018
IL-18	2.16448163	1.20371042	1.7981747	0.08654215
IL-21	-7.1015991	1.20371042	-5.8997571	7.42E-06
IFNy	-1.8106322	1.20371042	-1.5042091	0.14741957
TNFa	23.539892	1.20371042	19.5561088	5.87E-15
RANTES	0.16204596	1.20371042	0.13462205	0.89419264
SCF*Flt3L	7.10842437	1.6480454	4.31324549	0.00030721
SCF*TNFa	-8.3335326	1.56078911	-5.3393072	2.70E-05
Flt3L*IL-1a	10.7766857	1.69421559	6.36087034	2.63E-06
IL-1b*IL-18	-4.7305962	1.39240836	-3.3974202	0.00271445
IL-1b*TNFa	-9.4410142	1.36780148	-6.9023278	8.06E-07
IL-1b*RANTES	-4.4898134	1.60067235	-2.8049547	0.01061109
IL-3*IFNy	-2.5080706	1.71421754	-1.4630994	0.15824749
IL-3*RANTES	-3.6262683	1.70444908	-2.127531	0.04538772
IL-7*IL-7	-17.328283	4.41783964	-3.9223431	0.0007821

Supplementary Table 3 (Related to Figure 1). Linear regression model terms for CD7^{+} cells from day 0-7. Provided as raw values at the default precision in JMP 13 software that was used for regression.

Term	Scaled Estimate	Std Error	t Ratio	Prob>|t|
Intercept	38.2354304	2.28993593	16.6971617	1.03E-14
SCF	3.46255372	0.70658425	4.90041168	5.35E-05
Flt3L	0.7221513	0.70658425	1.0220314	0.31695825
IL-1a	0.93794616	0.70658425	1.32743712	0.19685369
IL-3	6.96508011	0.70658425	9.85739505	6.50E-10
IL-7	2.92510744	0.70658425	4.13978578	0.00036989
IL-15	-1.4546059	0.70658425	-2.0586447	0.05054079
IL-18	0.59658713	0.70658425	0.84432554	0.40682544
IFNγ	-1.4251344	0.70658425	-2.0169349	0.05502206
TNFa	17.1896859	0.70658425	24.3278644	2.01E-18
RANTES	-0.7447359	0.70658425	-1.0539945	0.3023814
SCF*IL-7	-6.1424113	0.95590818	-6.4257336	1.21E-06
SCF*IFNγ	-5.1511001	0.79602838	-6.4710006	1.08E-06
SCF*TNFa	-5.8116604	0.78933744	-7.3627071	1.33E-07
Flt3L*IFNγ	-2.8996081	0.85006097	-3.411059	0.00229435
IL-1a*IL-18	-2.0964354	0.81809125	-2.5625936	0.01708609
IL-1a*RANTES	4.4779813	0.85660531	5.22758994	2.34E-05
IFNγ*IFNγ	-9.5840397	2.41951521	-3.9611405	0.00058124

Supplementary Table 4 (Related to Figure 1). Linear regression model terms for CD7$^+$CD5$^+$ cells from day 0-7. Provided as raw values at the default precision in JMP 13 software that was used for regression.

Term	Scaled Estimate	Std Error	t Ratio	Prob>|t|
Intercept	88.2119145	7.44984267	11.8407755	1.65E-11
Block[1]	12.2212261	2.32187531	5.26351523	2.14E-05
Block[2]	-12.221226	2.32187531	-5.2635152	2.14E-05
SCF	11.8872455	2.09706254	5.66852217	7.74E-06
TPO	-3.5716481	2.09706254	-1.7031672	0.10145437
IL-3	16.6548214	2.09706254	7.94197648	3.59E-08
IL-15	-4.9546677	2.09706254	-2.3626705	0.02659002
IL-21	3.75240108	2.09706254	1.7893606	0.08618547
IFNγ	-10.561468	2.09706254	-5.036315	3.79E-05
TNFa	17.4650403	2.09706254	8.32833548	1.54E-08
CXCL12	0.98962735	2.09706254	0.47191123	0.64125256
SCF*IFNγ	-9.6348035	2.42995194	-3.9650181	0.00057558
TPO*IFNγ	-9.791543	2.52912493	-3.8715142	0.00072856
IL-3*IL-15	-9.9531221	2.5795491	-3.8584736	0.00075287
IL-3*IL-21	10.5685464	2.41458907	4.37695445	0.00020254
IL-3*CXCL12	-10.476515	2.66295626	-3.934167	0.00062217
IL-15*IL-21	-5.1613043	2.53626398	-2.0350028	0.05303928
IFNγ*TNFa	7.59653929	2.67356555	2.84135143	0.0090177
IL-15*IL-15	-52.338332	7.9333962	-6.5972165	8.00E-07

Supplementary Table 5 (Related to Figure 1). Linear regression model terms for total cells from day 7-14. Provided as raw values at the default precision in JMP 13 software that was used for regression.

Term	Scaled Estimate	Std Error	t Ratio	Prob>|t|
Intercept	-0.4294464	1.05235746	-0.4080804	0.68655584
Block[1]	-5.0276265	0.31540451	-15.940249	6.16E-15
Block[2]	5.02762648	0.31540451	15.9402491	6.16E-15
SCF	7.19690786	0.32688977	22.0163141	2.45E-18
Flt3L	1.19713814	0.32688977	3.6622074	0.00112089
IL-1a	1.37285996	0.32688977	4.1997642	0.00027722
IL-3	3.17619827	0.32688977	9.71641988	3.85E-10
IL-6	-1.4756853	0.32688977	-4.5143208	0.00012114
IL-7	1.65815678	0.32688977	5.07252576	2.78E-05
IL-18	0.55791221	0.32688977	1.70672886	0.09979047
IL-21	-0.759394	0.32688977	-2.3230888	0.02826392
IFNγ	-0.7815329	0.32688977	-2.3908148	0.02434207
TNFa	2.91092812	0.32688977	8.90492264	2.24E-09
RANTES	-1.454269	0.32688977	-4.4488054	0.00014398
Flt3L*RANTES	0.76822075	0.342659	2.24193955	0.03371702
IL-3*RANTES	0.58978318	0.342659	1.72119568	0.09709567
IL-21*IL-21	4.54250539	1.11120245	4.08791882	0.00037159

Supplementary Table 6 (Related to Figure 1). Linear regression model terms for $CD7^+$ cells from day 7-14. Provided as raw values at the default precision in JMP 13 software that was used for regression.

Term	Scaled Estimate	Std Error	t Ratio	Prob>|t|
Intercept	8.46044005	1.27962456	6.61165805	1.94E-06
Block[1]	-5.0928099	0.43910176	-11.598245	2.48E-10
Block[2]	5.09280988	0.43910176	11.5982451	2.48E-10
SCF	3.79869897	0.39057606	9.72588788	5.04E-09
Flt3L	1.13930796	0.39057606	2.91699383	0.0085242
TPO	0.79111712	0.39057606	2.0255136	0.05636689
IL-1a	1.67013143	0.39057606	4.27607218	0.00036892
IL-1b	1.94766572	0.39057606	4.986649	7.09E-05
IL-3	2.81688755	0.39057606	7.21213572	5.56E-07
IL-6	-0.8633749	0.39057606	-2.2105167	0.0388855
IL-7	3.73510864	0.39057606	9.56307625	6.67E-09
IL-15	0.55677285	0.39057606	1.4255171	0.16942901
IL-21	-0.4414777	0.39057606	-1.1303246	0.2717132
IFNγ	-1.5284321	0.39057606	-3.9132765	0.00086162
TNFa	4.87670456	0.39057606	12.485928	6.72E-11
RANTES	-0.9652458	0.39057606	-2.4713389	0.02257089
SCF*TNFa	-0.9089791	0.47216179	-1.9251432	0.06855202
TPO*IL-1b	3.23140275	0.50087236	6.4515493	2.72E-06
TPO*IL-21	-1.8752665	0.48234336	-3.8878249	0.00091441
IL-1a*TNFa	2.00931716	0.44444679	4.52093976	0.00020836
IL-3*IFNγ	0.86804295	0.55702447	1.55835695	0.13483258
IL-7*TNFa	2.1101213	0.51227722	4.11910037	0.0005325
IL-3*IL-3	-3.7070962	1.3534027	-2.7390932	0.01264601

Supplementary Table 7 (Related to Figure 1). Linear regression model terms for $CD7^{+}CD5^{+}$ cells from day 7-14. Provided as raw values at the default precision in JMP 13 software that was used for regression.

Term	Scaled Estimate	Std Error	t Ratio	Prob>|t|
Intercept	1.41785632	0.17358573	8.16804634	2.75E-07
Block[1]	-0.5518726	0.05768997	-9.5661803	2.95E-08
Block[2]	0.55187261	0.05768997	9.5661803	2.95E-08
SCF	0.69330822	0.04658126	14.8838456	3.50E-11
Flt3L	0.15000886	0.04658126	3.22036967	0.00502245
TPO	0.09709329	0.04658126	2.08438542	0.05252144
IL-1a	0.15465599	0.04658126	3.32013345	0.00405013
IL-1b	0.08215625	0.04658126	1.76371908	0.0957415
IL-3	0.33033842	0.04658126	7.0916597	1.81E-06
IL-6	-0.103375	0.04658126	-2.2192409	0.04036517
IL-7	0.45819466	0.04658126	9.83645997	1.97E-08
IL-15	-0.1457697	0.04658126	-3.129364	0.00610763
IL-18	-0.1329391	0.04658126	-2.8539173	0.01098298
IL-21	-0.1124967	0.04658126	-2.4150635	0.02728165
IFNγ	-0.5120009	0.04658126	-10.991565	3.81E-09
TNFa	0.58937963	0.04658126	12.6527209	4.46E-10
RANTES	-0.1170682	0.04658126	-2.513204	0.02233552
CXCL12	0.00768674	0.04658126	0.16501783	0.87087631
IL-1a*IL-7	0.33797203	0.07599821	4.44710524	0.0003536
IL-1b*TNFa	-0.2325363	0.05320178	-4.3708372	0.00041646
IL-6*IFNγ	0.162921	0.06287869	2.59103686	0.01902942
IL-7*TNFa	0.25344169	0.05413476	4.68168096	0.00021438
IL-18*TNFa	0.17736364	0.05940917	2.98545924	0.00830821
TNFa*CXCL12	-0.3137906	0.05479505	-5.7266228	2.47E-05
IL-1b*IL-1b	-0.4097009	0.21091751	-1.9424697	0.06883406
IL-18*IL-18	-1.6892753	0.20882721	-8.0893451	3.14E-07

Supplementary Table 8 (Related to Supplementary Figure 1). Factor concentrations for screening validation experiments.

	Concentration (ng/ml)		
**Factor**	**-1**	**0**	**1**
SCF	3	30	300
Flt3L	3	30	300
TPO	3	30	300
IL-1a	0.5	5	50
IL-1b	0.5	5	50
IL-3	1	10	100
IL-7	3	30	300
IL-21	1	10	100
TNFa	1	10	100
CXCL12	3	30	300

Supplementary Table 9 (Related to Supplementary Figure 1). Linear regression model terms for total cells from day 0-7. Provided as raw values at the default precision in JMP 13 software that was used for regression.

Term	Scaled Estimate	Std Error	t Ratio	Prob>|t|
Intercept	421.7535	15.79248	26.70597	1.57E-16
SCF	49.50128	6.087245	8.131967	1.31E-07
Flt3L	19.94848	6.087245	3.277095	0.003965
IL-1a	-23.8007	6.087245	-3.90992	0.000941
IL-1b	23.58617	6.087245	3.874688	0.00102
IL-3	3.677086	6.087245	0.604064	0.552946
IL-7	-12.4957	6.087245	-2.05277	0.054127
IL-21	-26.9593	6.087245	-4.42882	0.000288
TNFa	31.11326	6.087245	5.111221	6.21E-05
CXCL12	9.255202	6.087245	1.520425	0.144872
Flt3L*Flt3L	-96.1159	21.97905	-4.37307	0.000327
IL-1a*IL-1a	63.68215	22.61573	2.815835	0.011036
TNFa*TNFa	-96.2695	21.99432	-4.37702	0.000324
Flt3L*IL-3	29.90263	7.14127	4.187299	0.0005
Flt3L*CXCL12	15.38151	6.572035	2.340448	0.030323
IL-1a*IL-3	-29.4113	6.606348	-4.45197	0.000273
IL-21*TNFa	13.91181	7.072824	1.966939	0.063968

Supplementary Table 10 (Related to Supplementary Figure 1). Linear regression model terms for $CD7^+$ cells from day 0-7. Provided as raw values at the default precision in JMP 13 software that was used for regression.

Term	Scaled Estimate	Std Error	t Ratio	Prob>|t|
Intercept	324.0167	11.23244	28.84652	1.6E-16
SCF	23.20677	4.231553	5.48422	3.3E-05
Flt3L	18.73727	4.231553	4.427989	0.000325
IL-1a	-17.9357	4.231553	-4.23856	0.000494
IL-1b	19.39555	4.231553	4.583554	0.00023
IL-3	4.937447	4.231553	1.166817	0.258511
IL-7	-5.87237	4.231553	-1.38776	0.182152
IL-21	-7.96603	4.231553	-1.88253	0.076031
TNFa	34.63903	4.231553	8.18589	1.76E-07
CXCL12	8.542467	4.231553	2.018754	0.058663
Flt3L*Flt3L	-37.2207	15.33467	-2.42723	0.025934
IL-1a*IL-1a	50.16291	16.83598	2.979506	0.008035
IL-3*IL-3	-25.7467	14.75829	-1.74456	0.098113
TNFa*TNFa	-92.0613	17.63344	-5.22083	5.77E-05
SCF*IL-3	7.680827	5.343498	1.437416	0.167757
Flt3L*IL-3	16.3621	4.998596	3.273339	0.004223
Flt3L*CXCL12	13.04087	4.75419	2.743027	0.01337
IL-1a*IL-3	-26.431	4.705941	-5.61652	2.49E-05

Supplementary Table 11 (Related to Supplementary Figure 1). Linear regression model terms for $CD7^{+}CD5^{+}$ cells from day 0-7. Provided as raw values at the default precision in JMP 13 software that was used for regression.

Term	Scaled Estimate	Std Error	t Ratio	Prob>|t|
Intercept	120.3282	4.597319	26.17357	1.25E-12
Block[1]	-13.3621	4.049695	-3.29953	0.005753
Block[2]	13.36209	4.049695	3.29953	0.005753
SCF	16.45338	1.729571	9.512984	3.2E-07
Flt3L	7.705694	1.729571	4.455263	0.000648
IL-1a	-10.2907	1.729571	-5.94986	4.83E-05
IL-1b	9.151321	1.729571	5.291093	0.000146
IL-3	4.077907	1.729571	2.357756	0.03472
IL-7	-3.96261	1.729571	-2.29109	0.039303
IL-21	-4.48671	1.729571	-2.59412	0.022253
TNFa	1.505027	1.729571	0.870173	0.399988
CXCL12	3.460036	1.729571	2.000517	0.066779
Flt3L*Flt3L	-13.4037	15.08061	-0.8888	0.390259
IL-7*IL-7	-33.4941	14.42399	-2.32211	0.037103
SCF*IL-1b	-6.12621	2.798603	-2.18902	0.047444
SCF*IL-21	-10.3563	5.692225	-1.81937	0.091953
SCF*TNFa	-9.03616	2.24282	-4.02893	0.001432
IL-1a*IL-1b	12.40104	2.364721	5.24419	0.000158
IL-1a*IL-3	-7.47573	2.702475	-2.76625	0.016032
IL-1a*TNFa	-14.3917	7.582211	-1.89809	0.080111
IL-1b*IL-3	-1.94854	3.671769	-0.53068	0.604588
IL-1b*IL-7	14.24745	7.460918	1.909612	0.078498
IL-1b*CXCL12	2.759172	3.646236	0.756718	0.462713
IL-3*TNFa	-1.55565	2.754867	-0.56469	0.581897

Supplementary Table 12 (Related to Supplementary Figure 1). Linear regression model terms for total cells from day 7-14. Provided as raw values at the default precision in JMP 13 software that was used for regression.

Term	Scaled Estimate	Std Error	t Ratio	Prob>|t|
Intercept	237.825	3.770277	63.07892	1.42E-22
Block[1]	12.96798	1.934785	6.702544	2.76E-06
Block[2]	-12.968	1.934785	-6.70254	2.76E-06
SCF	20.31032	1.44464	14.05909	3.79E-11
IL-1b	0.319537	1.44464	0.221188	0.827436
IL-3	8.790551	1.44464	6.084943	9.47E-06
IL-7	72.43007	1.44464	50.13711	8.64E-21
IL-21	-10.2499	1.44464	-7.09516	1.3E-06
TNFa	15.64402	1.44464	10.82901	2.59E-09
CXCL12	4.857228	1.44464	3.362241	0.00347
IL-1b*IL-3	-2.55604	1.807759	-1.41393	0.174447
IL-1b*CXCL12	-9.80432	1.805492	-5.43028	3.69E-05
IL-3*CXCL12	-3.30956	1.874916	-1.76518	0.094493
IL-7*IL-21	-7.84978	1.619706	-4.84643	0.00013
IL-7*TNFa	15.34255	1.820535	8.427498	1.16E-07
IL-7*CXCL12	2.905552	1.626214	1.786698	0.09084
TNFa*CXCL12	3.862878	1.70058	2.271506	0.03562
SCF*SCF	-19.3747	5.094722	-3.8029	0.001303
IL-7*IL-7	-65.1195	4.913722	-13.2526	1E-10

Supplementary Table 13 (Related to Supplementary Figure 1). Linear regression model terms for CD7$^+$ cells from day 7-14. Provided as raw values at the default precision in JMP 13 software that was used for regression.

Term	Scaled Estimate	Std Error	t Ratio	Prob>|t|
Intercept	213.0429	4.340803	49.07915	1.77E-21
Block[1]	8.018208	2.394469	3.348638	0.003374
Block[2]	-8.01821	2.394469	-3.34864	0.003374
SCF	8.842854	1.660564	5.325211	3.87E-05
IL-3	7.327362	1.660564	4.412574	0.000299
IL-7	83.47695	1.660564	50.27024	1.12E-21
IL-21	-10.2832	1.660564	-6.1926	5.98E-06
TNFa	12.86034	1.660564	7.744562	2.7E-07
CXCL12	4.224562	1.660564	2.544052	0.019807
SCF*IL-7	7.063645	2.466983	2.863273	0.009949
SCF*TNFa	-3.81721	1.876099	-2.03465	0.056084
SCF*CXCL12	-5.59685	2.105053	-2.65877	0.015506
IL-7*IL-21	-13.3368	1.967794	-6.77753	1.79E-06
IL-7*TNFa	13.38199	2.162249	6.188922	6.03E-06
IL-7*CXCL12	6.130731	1.926027	3.183098	0.004896
IL-3*IL-3	-15.4358	9.207936	-1.67636	0.110045
IL-7*IL-7	-55.4034	6.933754	-7.99039	1.71E-07
TNFa*TNFa	-23.8174	7.084963	-3.36168	0.003276

Supplementary Table 14 (Related to Supplementary Figure 1). Linear regression model terms for $CD7^+CD5^+$ cells from day 7-14. Provided as raw values at the default precision in JMP 13 software that was used for regression.

Term	Scaled Estimate	Std Error	t Ratio	Prob>|t|
Intercept	237.825	3.770277	63.07892	1.42E-22
Block[1]	12.96798	1.934785	6.702544	2.76E-06
Block[2]	-12.968	1.934785	-6.70254	2.76E-06
SCF	20.31032	1.44464	14.05909	3.79E-11
IL-1b	0.319537	1.44464	0.221188	0.827436
IL-3	8.790551	1.44464	6.084943	9.47E-06
IL-7	72.43007	1.44464	50.13711	8.64E-21
IL-21	-10.2499	1.44464	-7.09516	1.3E-06
TNFa	15.64402	1.44464	10.82901	2.59E-09
CXCL12	4.857228	1.44464	3.362241	0.00347
IL-1b*IL-3	-2.55604	1.807759	-1.41393	0.174447
IL-1b*CXCL12	-9.80432	1.805492	-5.43028	3.69E-05
IL-3*CXCL12	-3.30956	1.874916	-1.76518	0.094493
IL-7*IL-21	-7.84978	1.619706	-4.84643	0.00013
IL-7*TNFa	15.34255	1.820535	8.427498	1.16E-07
IL-7*CXCL12	2.905552	1.626214	1.786698	0.09084
TNFa*CXCL12	3.862878	1.70058	2.271506	0.03562
SCF*SCF	-19.3747	5.094722	-3.8029	0.001303
IL-7*IL-7	-65.1195	4.913722	-13.2526	1E-10

Supplementary Table 15 (Related to Figure 4). Factor concentrations for RSM experiments.

	Scaled factor level					
**Factor**	**-2.366**	**-1**	**0**	**1**	**2.366**	
SCF	0.52	2.86	10.00	35.00	193.76	ng/ml
Flt3L	0.52	2.86	10.00	35.00	193.76	ng/ml
IL-3	0.05	0.29	1.00	3.50	19.38	ng/ml
IL-7	0.52	2.86	10.00	35.00	193.76	ng/ml
TNFa	0.03	0.14	0.50	1.75	9.69	ng/ml
CXCL12	0.52	2.86	10.00	35.00	193.76	ng/ml

Supplementary Table 16 (Related to Figure 4). Linear regression model terms for ProT-cells from day 0-7. Provided as raw values at the default precision in JMP 14 software that was used for regression.

Term	Estimate	Std Error	t Ratio	Prob>|t|
Intercept	71.6797471	1.67416813	42.82	<.0001
SCF	9.9138704	1.85717241	5.34	<.0001
IL-3	15.8415123	1.85717241	8.53	<.0001
TNFa	32.033221	1.85717241	17.25	<.0001
SCF*TNFa	7.19163125	2.15783934	3.33	0.0017
IL-3*TNFa	10.4289214	2.15783934	4.83	<.0001
Block[1]	7.6578892	2.29917013	3.33	0.0017
Block[2]	-0.2290307	2.32742038	-0.1	0.922

Supplementary Table 17 (Related to Figure 4). Linear regression model terms for CD4ISP cells from day 0-7. Provided as raw values at the default precision in JMP 14 software that was used for regression.

Term	Estimate	Std Error	t Ratio	Prob>|t|
Intercept	103.61767	2.0413071	50.76	<.0001
SCF	16.8207395	2.26444355	7.43	<.0001
IL-3	13.8267354	2.26444355	6.11	<.0001
TNFa	17.5943395	2.26444355	7.77	<.0001
SCF*TNFa	4.70545012	2.63104565	1.79	0.0803
IL-3*TNFa	2.48957783	2.63104565	0.95	0.349
Block[1]	14.2788352	2.80336975	5.09	<.0001
Block[2]	1.7624824	2.83781518	0.62	0.5376

Supplementary Table 18 (Related to Figure 4). Linear regression model terms for early DP cells from day 0-7. Provided as raw values at the default precision in JMP 14 software that was used for regression.

Term	Estimate	Std Error	t Ratio	Prob>|t|
Intercept	23.5976571	0.51419461	45.89	<.0001
SCF	1.23598546	0.57040152	2.17	0.0355
IL-3	2.32434246	0.57040152	4.07	0.0002
TNFa	3.15202229	0.57040152	5.53	<.0001
SCF*TNFa	0.1023632	0.66274667	0.15	0.8779
IL-3*TNFa	0.38530373	0.66274667	0.58	0.5638
Block[1]	4.96773292	0.70615421	7.03	<.0001
Block[2]	0.5066865	0.71483084	0.71	0.482

Supplementary Table 19 (Related to Figure 4). Linear regression model terms for proT-cells from day 7-14. Provided as raw values at the default precision in JMP 14 software that was used for regression.

Term	Estimate	Std Error	t Ratio	Prob>|t|
Intercept	291.628979	6.14680328	47.44	<.0001
SCF	17.6115579	3.6017748	4.89	<.0001
Flt3L	-4.0484279	3.6017748	-1.12	0.2677
IL-7	174.729196	5.32166436	32.83	<.0001
TNFa	-0.0298286	3.6017748	-0.01	0.9934
CXCL12	12.0255116	3.6017748	3.34	0.0018
TNFa*CXCL12	-26.458075	4.18488415	-6.32	<.0001
SCF*SCF	-4.6428883	3.06379363	-1.52	0.1375
Flt3L*Flt3L	-13.788429	3.06379363	-4.5	<.0001
IL-7*IL-7	-12.485266	3.06379363	-4.08	0.0002
CXCL12*CXCL12	-8.8235862	3.06379363	-2.88	0.0064
Block[1]	39.2710377	4.45904662	8.81	<.0001
Block[2]	5.06404619	4.52376056	1.12	0.2696
IL-7*IL-7*IL-7	-21.397621	1.78672811	-11.98	<.0001

Supplementary Table 20 (Related to Figure 4). Linear regression model terms for CD4ISP cells from day 7-14. Provided as raw values at the default precision in JMP 14 software that was used for regression.

Term	Estimate	Std Error	t Ratio	Prob>|t|
Intercept	133.739148	4.0060544	33.38	<.0001
SCF	24.729668	2.3473837	10.53	<.0001
Flt3L	-2.3644042	2.3473837	-1.01	0.3199
IL-7	23.7706908	3.46828684	6.85	<.0001
TNFa	5.02661963	2.3473837	2.14	0.0384
CXCL12	5.12097499	2.3473837	2.18	0.0351
TNFa*CXCL12	-5.9615814	2.72741339	-2.19	0.0347
SCF*SCF	-3.0420872	1.99676538	-1.52	0.1355
Flt3L*Flt3L	-7.4211583	1.99676538	-3.72	0.0006
IL-7*IL-7	-9.420782	1.99676538	-4.72	<.0001
CXCL12*CXCL12	-5.5856195	1.99676538	-2.8	0.0079
Block[1]	19.3786765	2.90609323	6.67	<.0001
Block[2]	0.11294902	2.94826922	0.04	0.9696
IL-7*IL-7*IL-7	-2.4283895	1.16446382	-2.09	0.0435

Supplementary Table 21 (Related to Figure 4). Linear regression model terms for early DP cells from day 7-14. Provided as raw values at the default precision in JMP 14 software that was used for regression.

Term	Estimate	Std Error	t Ratio	Prob>|t|
Intercept	27.4192258	1.02921097	26.64	<.0001
SCF	4.99336479	0.60307545	8.28	<.0001
Flt3L	-1.049385	0.60307545	-1.74	0.0895
IL-7	9.67417562	0.89105102	10.86	<.0001
TNFa	0.45534857	0.60307545	0.76	0.4546
CXCL12	1.29563789	0.60307545	2.15	0.0378
TNFa*CXCL12	-2.2437249	0.70071035	-3.2	0.0027
SCF*SCF	-0.1581914	0.51299673	-0.31	0.7594
Flt3L*Flt3L	-1.3805223	0.51299673	-2.69	0.0103
IL-7*IL-7	-1.5753906	0.51299673	-3.07	0.0038
CXCL12*CXCL12	-1.3714581	0.51299673	-2.67	0.0108
Block[1]	3.71456732	0.74661568	4.98	<.0001
Block[2]	0.35401123	0.75745127	0.47	0.6428
IL-7*IL-7*IL-7	-1.1331093	0.29916691	-3.79	0.0005

Supplementary Table 22 (Related to Figure 4). Linear regression model terms for CD4ISP cells from day 14-21. Provided as raw values at the default precision in JMP 14 software that was used for regression.

Term	Estimate	Std Error	t Ratio	Prob>|t|
Intercept	120.237143	2.96960579	40.49	<.0001
SCF	27.0731414	2.63666224	10.27	<.0001
Flt3L	-1.44686	2.63666224	-0.55	0.5862
IL-3	3.88158144	2.63666224	1.47	0.1488
IL-7	30.4154464	3.89569926	7.81	<.0001
TNFa	4.03558101	2.63666224	1.53	0.1337
CXCL12	3.03577086	2.63666224	1.15	0.2564
Flt3L*IL-3	-0.4743265	3.06352468	-0.15	0.8777
SCF*IL-7	2.81594141	3.06352468	0.92	0.3635
TNFa*CXCL12	-7.4490183	3.06352468	-2.43	0.0196
IL-7*IL-7	-6.8304829	2.23572417	-3.06	0.004
Block[1]	21.452036	3.26418535	6.57	<.0001
Block[2]	3.90525978	3.30587154	1.18	0.2445
IL-7*IL-7*IL-7	-3.0163804	1.30796588	-2.31	0.0264

Supplementary Table 23 (Related to Figure 4). Linear regression model terms for early DP cells from day 14-21. Provided as raw values at the default precision in JMP 14 software that was used for regression.

Term	Estimate	Std Error	t Ratio	Prob>|t|
Intercept	52.9982187	1.2782537	41.46	<.0001
SCF	10.4819647	1.13493962	9.24	<.0001
Flt3L	-1.0781755	1.13493962	-0.95	0.3478
IL-3	1.0539978	1.13493962	0.93	0.3586
IL-7	16.5635165	1.67688655	9.88	<.0001
TNFa	2.66784135	1.13493962	2.35	0.0238
CXCL12	2.38344237	1.13493962	2.1	0.0421
Flt3L*IL-3	0.43283012	1.31868068	0.33	0.7445
SCF*IL-7	1.56543433	1.31868068	1.19	0.2422
TNFa*CXCL12	-2.4524409	1.31868068	-1.86	0.0703
IL-7*IL-7	-2.3078868	0.9623576	-2.4	0.0212
Block[1]	10.7118481	1.40505418	7.62	<.0001
Block[2]	1.85193911	1.42299781	1.3	0.2006
IL-7*IL-7*IL-7	-1.8447401	0.56300814	-3.28	0.0022

Supplementary Table 24 (Related to Figure 4). Linear regression model terms for late DP cells from day 14-21. Provided as raw values at the default precision in JMP 14 software that was used for regression.

Term	Estimate	Std Error	t Ratio	Prob>|t|
Intercept	9.70567708	0.36147161	26.85	<.0001
SCF	0.956464	0.32094446	2.98	0.0049
Flt3L	-0.273237	0.32094446	-0.85	0.3996
IL-3	0.16991784	0.32094446	0.53	0.5994
IL-7	3.62133847	0.4741992	7.64	<.0001
TNFa	-0.28819	0.32094446	-0.9	0.3746
CXCL12	0.2827937	0.32094446	0.88	0.3835
Flt3L*IL-3	-1.1503788	0.37290377	-3.08	0.0037
SCF*IL-7	1.03096742	0.37290377	2.76	0.0086
TNFa*CXCL12	-0.9698463	0.37290377	-2.6	0.013
IL-7*IL-7	-0.7988181	0.27214077	-2.94	0.0055
Block[1]	1.52363388	0.39732894	3.83	0.0004
Block[2]	0.42112253	0.40240314	1.05	0.3016
IL-7*IL-7*IL-7	-0.6375127	0.15921054	-4	0.0003

Supplementary Table 25 (Related to Figure 4). Linear regression model terms for CD8SP cells from day 14-21. Provided as raw values at the default precision in JMP 14 software that was used for regression.

Term	Estimate	Std Error	t Ratio	Prob>|t|
Intercept	11.5826367	0.44596741	25.97	<.0001
SCF	-0.5634655	0.39596685	-1.42	0.1625
Flt3L	-0.0394127	0.39596685	-0.1	0.9212
IL-3	-0.0839281	0.39596685	-0.21	0.8332
IL-7	5.35939537	0.58504564	9.16	<.0001
TNFa	-0.4133108	0.39596685	-1.04	0.3028
CXCL12	0.10208908	0.39596685	0.26	0.7979
Flt3L*IL-3	-0.1256408	0.4600719	-0.27	0.7862
SCF*IL-7	1.20911486	0.4600719	2.63	0.0121
TNFa*CXCL12	-0.2820543	0.4600719	-0.61	0.5433
IL-7*IL-7	-0.1251572	0.33575505	-0.37	0.7113
Block[1]	3.0954772	0.49020658	6.31	<.0001
Block[2]	0.6788045	0.4964669	1.37	0.1792
IL-7*IL-7*IL-7	-0.7262834	0.1964268	-3.7	0.0007

Supplementary Table 26 (Related to Figure 4). Linear regression model terms for CD4ISP cells from day 21-28. Provided as raw values at the default precision in JMP 14 software that was used for regression.

Term	Estimate	Std Error	t Ratio	Prob>|t|
Intercept	103.164804	2.38017518	43.34	<.0001
SCF	15.857939	1.56543736	10.13	<.0001
IL-7	55.5635627	2.31295199	24.02	<.0001
TNFa	-3.6147244	1.56543736	-2.31	0.0261
CXCL12	0.71757033	1.56543736	0.46	0.6491
SCF*IL-7	14.8698393	1.81887385	8.18	<.0001
TNFa*CXCL12	-3.485291	1.81887385	-1.92	0.0623
SCF*SCF	-3.1972287	1.33007366	-2.4	0.0208
IL-7*IL-7	-8.1344651	1.33007366	-6.12	<.0001
TNFa*TNFa	1.03273376	1.33007366	0.78	0.4419
Block[1]	9.88119172	1.93802454	5.1	<.0001
Block[2]	-0.4409151	1.96491797	-0.22	0.8236
IL-7*IL-7*IL-7	-8.2384403	0.77656464	-10.61	<.0001

Supplementary Table 27 (Related to Figure 4). Linear regression model terms for early DP cells from day 21-28. Provided as raw values at the default precision in JMP 14 software that was used for regression.

Term	Estimate	Std Error	t Ratio	Prob>|t|
Intercept	86.6349143	2.0179206	42.93	<.0001
SCF	19.1067611	1.32718311	14.4	<.0001
IL-7	35.4637233	1.96092856	18.09	<.0001
TNFa	1.84469728	1.32718311	1.39	0.172
CXCL12	0.21150468	1.32718311	0.16	0.8742
SCF*IL-7	12.3528251	1.54204743	8.01	<.0001
TNFa*CXCL12	-2.3933864	1.54204743	-1.55	0.1283
SCF*SCF	-1.3848479	1.12764097	-1.23	0.2264
IL-7*IL-7	-7.1153746	1.12764097	-6.31	<.0001
TNFa*TNFa	2.8558109	1.12764097	2.53	0.0152
Block[1]	10.9429091	1.64306379	6.66	<.0001
Block[2]	-0.8823612	1.66586413	-0.53	0.5992
IL-7*IL-7*IL-7	-5.6418604	0.65837414	-8.57	<.0001

Supplementary Table 28 (Related to Figure 4). Linear regression model terms for late DP cells from day 21-28. Provided as raw values at the default precision in JMP 14 software that was used for regression.

Term	Estimate	Std Error	t Ratio	Prob>|t|
Intercept	43.1526317	1.06544374	40.5	<.0001
SCF	6.50420244	0.70074063	9.28	<.0001
IL-7	16.6457211	1.03535246	16.08	<.0001
TNFa	-0.9553494	0.70074063	-1.36	0.1802
CXCL12	0.19378692	0.70074063	0.28	0.7835
SCF*IL-7	4.74860956	0.81418703	5.83	<.0001
TNFa*CXCL12	-2.4891032	0.81418703	-3.06	0.0039
SCF*SCF	-1.2644009	0.59538419	-2.12	0.0398
IL-7*IL-7	-3.3712565	0.59538419	-5.66	<.0001
TNFa*TNFa	1.81999508	0.59538419	3.06	0.0039
Block[1]	5.18271266	0.86752275	5.97	<.0001
Block[2]	-0.7477069	0.87956112	-0.85	0.4002
IL-7*IL-7*IL-7	-2.6706032	0.34761556	-7.68	<.0001

Supplementary Table 29 (Related to Figure 4). Linear regression model terms for CD8SP cells from day 21-28. Provided as raw values at the default precision in JMP 14 software that was used for regression.

Term	Estimate	Std Error	t Ratio	Prob>|t|
Intercept	32.2603708	0.7316126	44.09	<.0001
SCF	-0.2143526	0.48118042	-0.45	0.6583
IL-7	15.6124064	0.71094969	21.96	<.0001
TNFa	-1.1503109	0.48118042	-2.39	0.0215
CXCL12	-0.0120103	0.48118042	-0.02	0.9802
SCF*IL-7	-0.6638775	0.55908113	-1.19	0.2419
TNFa*CXCL12	-0.8405346	0.55908113	-1.5	0.1404
SCF*SCF	-1.0381491	0.40883488	-2.54	0.015
IL-7*IL-7	-2.4429343	0.40883488	-5.98	<.0001
TNFa*TNFa	0.68465812	0.40883488	1.67	0.1016
Block[1]	2.72608001	0.59570538	4.58	<.0001
Block[2]	-0.395117	0.60397182	-0.65	0.5166
IL-7*IL-7*IL-7	-2.2515367	0.23869859	-9.43	<.0001

Supplementary Table 30 (Related to Figure 4). Linear regression model terms for CD4ISP cells from day 28-35. Provided as raw values at the default precision in JMP 14 software that was used for regression.

Term	Estimate	Std Error	t Ratio	Prob>|t|
Intercept	53.5754183	0.75178221	71.26	<.0001
SCF	9.32179642	0.73054966	12.76	<.0001
IL-7	4.50674811	0.73054966	6.17	<.0001
TNFa	-4.8876511	0.49444594	-9.89	<.0001
SCF*IL-7	1.81056326	0.57449427	3.15	0.003
IL-7*TNFa	-0.4952053	0.57449427	-0.86	0.3937
SCF*SCF	-1.3044505	0.42010593	-3.11	0.0034
IL-7*IL-7	-2.846959	0.42010593	-6.78	<.0001
TNFa*TNFa	-0.4528421	0.42010593	-1.08	0.2874
Block[1]	1.32662297	0.61212821	2.17	0.0361
Block[2]	0.07712607	0.62062254	0.12	0.9017
SCF*SCF*SCF	-0.8449201	0.24527921	-3.44	0.0013
IL-7*IL-7*IL-7	0.23670069	0.24527921	0.97	0.3402

Supplementary Table 31 (Related to Figure 4). Linear regression model terms for early DP cells from day 28-35. Provided as raw values at the default precision in JMP 14 software that was used for regression.

Term	Estimate	Std Error	t Ratio	Prob>|t|
Intercept	71.5992903	0.99390129	72.04	<.0001
SCF	15.5347015	0.96583058	16.08	<.0001
IL-7	3.66666305	0.96583058	3.8	0.0005
TNFa	-7.6838777	0.65368727	-11.75	<.0001
SCF*IL-7	1.65981404	0.75951598	2.19	0.0346
IL-7*TNFa	-1.112947	0.75951598	-1.47	0.1505
SCF*SCF	-1.4944678	0.55540531	-2.69	0.0103
IL-7*IL-7	-3.0759031	0.55540531	-5.54	<.0001
TNFa*TNFa	-0.6824142	0.55540531	-1.23	0.2262
Block[1]	2.23170456	0.80927031	2.76	0.0087
Block[2]	0.21447548	0.82050033	0.26	0.7951
SCF*SCF*SCF	-1.7914292	0.32427386	-5.52	<.0001
IL-7*IL-7*IL-7	0.79880273	0.32427386	2.46	0.0181

Supplementary Table 32 (Related to Figure 4). Linear regression model terms for late DP cells from day 28-35. Provided as raw values at the default precision in JMP 14 software that was used for regression.

Term	Estimate	Std Error	t Ratio	Prob>|t|
Intercept	52.3310823	0.83554856	62.63	<.0001
SCF	12.1459767	0.8119502	14.96	<.0001
IL-7	1.34598688	0.8119502	1.66	0.105
TNFa	-4.615058	0.54953894	-8.4	<.0001
SCF*IL-7	0.96339826	0.63850655	1.51	0.139
IL-7*TNFa	-1.7068442	0.63850655	-2.67	0.0107
SCF*SCF	-0.9861832	0.46691569	-2.11	0.0408
IL-7*IL-7	-1.6067212	0.46691569	-3.44	0.0013
TNFa*TNFa	-0.1688805	0.46691569	-0.36	0.7194
Block[1]	2.82127714	0.6803338	4.15	0.0002
Block[2]	0.33549963	0.6897746	0.49	0.6293
SCF*SCF*SCF	-1.1632524	0.27260912	-4.27	0.0001
IL-7*IL-7*IL-7	0.68930846	0.27260912	2.53	0.0154

Supplementary Table 33 (Related to Figure 4). Linear regression model terms for CD8SP cells from day 28-35. Provided as raw values at the default precision in JMP 14 software that was used for regression.

Term	Estimate	Std Error	t Ratio	Prob>|t|
Intercept	27.7659427	0.35325225	78.6	<.0001
SCF	1.11467219	0.34327536	3.25	0.0023
IL-7	1.14175554	0.34327536	3.33	0.0019
TNFa	-2.3960274	0.23233343	-10.31	<.0001
SCF*IL-7	0.04770922	0.26994705	0.18	0.8606
IL-7*TNFa	-0.2525654	0.26994705	-0.94	0.355
SCF*SCF	-0.3045144	0.19740207	-1.54	0.1306
IL-7*IL-7	-1.1775153	0.19740207	-5.97	<.0001
TNFa*TNFa	-0.8254243	0.19740207	-4.18	0.0001
Block[1]	0.52141125	0.28763073	1.81	0.0772
Block[2]	-1.602835	0.2916221	-5.5	<.0001
SCF*SCF*SCF	0.18998604	0.11525337	1.65	0.1069
IL-7*IL-7*IL-7	0.44170581	0.11525337	3.83	0.0004

Supplementary Table 34 (Related to Figure 4). Linear regression model terms for CD4ISP cells from day 35-42. Provided as raw values at the default precision in JMP 14 software that was used for regression.

Term	Estimate	Std Error	t Ratio	Prob>|t|
Intercept	15.0784693	0.24322662	61.99	<.0001
SCF	0.6406532	0.18301698	3.5	0.001
IL-7	0.71577519	0.18301698	3.91	0.0003
TNFa	-0.6432405	0.18301698	-3.51	0.001
IL-7*IL-7	-0.3023616	0.15533652	-1.95	0.0577
TNFa*TNFa	-0.2681457	0.15533652	-1.73	0.091
Block[1]	1.06565489	0.22657566	4.7	<.0001
Block[2]	0.17154972	0.22958879	0.75	0.4587

Supplementary Table 35 (Related to Figure 4). Linear regression model terms for early DP cells from day 35-42. Provided as raw values at the default precision in JMP 14 software that was used for regression.

Term	Estimate	Std Error	t Ratio	Prob>|t|
Intercept	35.9457562	0.67086437	53.58	<.0001
SCF	6.26474675	0.50479497	12.41	<.0001
IL-7	2.92834805	0.50479497	5.8	<.0001
TNFa	-4.2569464	0.50479497	-8.43	<.0001
IL-7*IL-7	-1.4741273	0.42844711	-3.44	0.0012
TNFa*TNFa	-1.2646811	0.42844711	-2.95	0.005
Block[1]	3.26100196	0.62493794	5.22	<.0001
Block[2]	0.3823033	0.63324869	0.6	0.549

Supplementary Table 36 (Related to Figure 4). Linear regression model terms for late DP cells from day 35-42. Provided as raw values at the default precision in JMP 14 software that was used for regression.

Term	Estimate	Std Error	t Ratio	Prob>|t|
Intercept	47.8080595	1.09075514	43.83	<.0001
SCF	11.8192798	0.82074371	14.4	<.0001
IL-7	3.42460859	0.82074371	4.17	0.0001
TNFa	-4.3786688	0.82074371	-5.34	<.0001
IL-7*IL-7	-1.7459706	0.69661008	-2.51	0.0158
TNFa*TNFa	-1.3026167	0.69661008	-1.87	0.0679
Block[1]	6.59695715	1.01608358	6.49	<.0001
Block[2]	1.85039497	1.02959599	1.8	0.0789

Supplementary Table 37 (Related to Figure 4). Linear regression model terms for late DP cells from day 35-42. Provided as raw values at the default precision in JMP 14 software that was used for regression.

Term	Estimate	Std Error	t Ratio	Prob>|t|
Intercept	32.2984565	0.39517481	81.73	<.0001
SCF	3.30658065	0.2973511	11.12	<.0001
IL-7	1.85584257	0.2973511	6.24	<.0001
TNFa	-2.9674504	0.2973511	-9.98	<.0001
IL-7*IL-7	-0.8202399	0.25237814	-3.25	0.0022
TNFa*TNFa	-0.4319561	0.25237814	-1.71	0.0937
Block[1]	2.38903337	0.3681217	6.49	<.0001
Block[2]	0.346024	0.37301717	0.93	0.3584

Supplementary Table 38 (Related to Figure 5). Three-stage optimum cytokine concentrations (ng/ml).

	Interval (days)	SCF	Flt3L	IL-3	IL-7	TNFα	CXCL12
**Stage I**	0-7	23.9	8.7	5.3	10.0	4.9	9.7
**Stage II**	7-21	120.5	8.0	1.2	44.5	0.4	14.8
**Stage III**	21-42	77.1	9.8	1.0	33.5	0.1	15.7

Supplementary Table 39. HSPC and proT-cell antibodies used in this study.

**Antigen**	**Fluorochrome**	**Clone**	**Cat. Num.**	**Vendor**
CD117 (c-Kit)	AlexaFluor-488	104D2	313233	Biolegend
CD123 (IL-3R)	BV421	9F5	562517	BD Biosciences
CD127 (IL-7R)	PE Cy7	A019D5	351320	Biolegend
CD14	FITC	M5E2	555397	BD Biosciences
CD33	FITC	HIM3-4	303304	Biolegend
CD34	PE	581	555822	BD Biosciences
CD34	APC	581	555824	BD Biosciences
CD38	PE	HIT2	555460	BD Biosciences
CD5	PE Cy7	UCHT2	25-0059-42	eBiosciences
CD56	BV605	NCAM16.2	562780	BD Biosciences
CD7	APC	M-T701	561604	BD Biosciences

Supplementary Table 40. T-cell maturation antibodies used in this study.

Antigen	Fluorochrome	Clone	Cat. Num.	Vendor
CD3	APC Cy7	SK7	557832	BD Biosciences
CD4	BB515	SK3	566912	BD Biosciences
CD7	APC	M-T701	561604	BD Biosciences
CD8α	BV605	SK1	564116	BD Biosciences
CD8β	PE Cy7	SIDI8BEE	25-5273-42	eBiosciences
TCRαβ	BV711	IP26	306740	Biolegend
CD45RA	BV786	HI100	563870	BD Biosciences
CD27	BB700	M-T271	566449	BD Biosciences
CD27	APC	M-T271	558664	BD Biosciences
CD28	PE-Cy5	CD28.2	555730	BD Biosciences
CD197 (CCR7)	PE-CF594	150503	562381	BD Biosciences

Supplementary Table 41. Invariant TCR antibodies used in this study.

Antigen	Fluorochrome	Clone	Cat. Num.	Vendor
CD3	APC Cy7	SK7	557832	BD Biosciences
TCR Vα7.2	PE	OF-5A12	566739	BD Biosciences
TCR Vα24-Jα18	APC	6B11	342908	Biolegend

Supplementary Table 42. T-cell cytokine antibodies used in this study.

Antigen	Fluorochrome	Clone	Cat. Num.	Vendor
CD3	APC Cy7	SK7	557832	BD Biosciences
IL-2	APC	MQ1-17H12	561054	BD Biosciences
IFNγ	PE	4S.B3	554552	BD Biosciences

Supplementary Table 43. Primer sequences used for qPCR.

Gene	Forward	Reverse
ACTB1	CATGTACGTTGCTATCCAGGC	CTCCTTAATGTCACGCACGAT
BCL11B	TCCAGCTACATTTGCACAACA	GCTCCAGGTAGATGCGGAAG
CEBPA	GGAGCTGAGATCCCGACA	TTCTAAGGACAGGCGTGGAG
DTX1	ATCGGAGAAGGCTCTACAGG	CGTCTGGCCTCCTTTCTAACT
E2a	CCGACTCCTACAGTGGGCTA	CGCTGACGTGTTCTCCTCG
GATA3	GTTGGCCTAAGGTGGTTGTG	ACAGGCTGCAGGAATAGGGA
HES1	CCTGTCATCCCCGTCTACAC	CACATGGAGTCCGCCGTAA
NOTCH1	GAGGCGTGGCAGACTATGC	CTTGTACTCCGTCAGCGTGA
SPI1	TGCAATGTCAAGGGAGGGGG	AAACCCTTCCATTTTGCACGC
TCF7	TGCACATGCAGCTATACCCAG	TGGTGGATTCTTGGTGCTTTTC
